# Characterization of Live-Attenuated Powassan Virus Vaccine Candidates Identifies an Efficacious Prime-Boost Strategy for Mitigating Powassan Virus Disease in a Murine Model

**DOI:** 10.3390/vaccines11030612

**Published:** 2023-03-08

**Authors:** Andrew M. Cheung, Elaine Z. Yip, Alison W. Ashbrook, Niluka Goonawardane, Corrine Quirk, Charles M. Rice, Margaret R. MacDonald, Hans-Heinrich Hoffmann

**Affiliations:** 1 Laboratory of Virology and Infectious Disease, The Rockefeller University, New York, NY 10065, USA; 2 Nuffield Department of Medicine, Peter Medawar Building for Pathogen Research, University of Oxford, Oxford OX1 3SY, UK

**Keywords:** Powassan virus, deer tick virus, live-attenuated vaccine, yellow fever 17D virus vaccine, CpG and UpA dinucleotides, zinc finger antiviral protein (ZAP)

## Abstract

Powassan virus (POWV) is an emerging tick-borne virus and cause of lethal encephalitis in humans. The lack of treatment or prevention strategies for POWV disease underscores the need for an effective POWV vaccine. Here, we took two independent approaches to develop vaccine candidates. First, we recoded the POWV genome to increase the dinucleotide frequencies of CpG and UpA to potentially attenuate the virus by raising its susceptibility to host innate immune factors, such as the zinc-finger antiviral protein (ZAP). Secondly, we took advantage of the live-attenuated yellow fever virus vaccine 17D strain (YFV-17D) as a vector to express the structural genes pre-membrane (prM) and envelope (E) of POWV. The chimeric YFV-17D-POWV vaccine candidate was further attenuated for in vivo application by removing an N-linked glycosylation site within the nonstructural protein (NS)1 of YFV-17D. This live-attenuated chimeric vaccine candidate significantly protected mice from POWV disease, conferring a 70% survival rate after lethal challenge when administered in a homologous two-dose regimen. Importantly, when given in a heterologous prime-boost vaccination scheme, in which vaccination with the initial chimeric virus was followed by a protein boost with the envelope protein domain III (EDIII), 100% of the mice were protected without showing any signs of morbidity. Combinations of this live-attenuated chimeric YFV-17D-POWV vaccine candidate with an EDIII protein boost warrant further studies for the development of an effective vaccine strategy for the prevention of POWV disease.

## 1. Introduction

Powassan virus (POWV) is an emerging tick-borne virus found primarily in North America and in the Russian Far East. POWV is a member of the *Flaviviridae*, a family of enveloped, positive-stranded RNA viruses that includes tick-borne encephalitis virus (TBEV) and the mosquito-borne viruses Zika (ZIKV), dengue (DENV), Japanese encephalitis (JEV), West Nile (WNV), and yellow fever (YFV). Viruses of this family are maintained in nature through transmission cycles between vector species (ticks or mosquitoes) and vertebrate reservoirs, and are well known to cause significant human morbidity and mortality worldwide. Symptomatic POWV infection results in a febrile illness that can progress to neuroinvasive disease presenting as encephalitis, meningoencephalitis, or meningitis. This is fatal in ~10% of the cases [1] and a high proportion (>50%) of survivors are at risk for long-term sequelae. Moreover, there is an increasing concern for the potential of POWV to become a significant public health issue following the rise in the incidence of tick-related human disease in North America [2]. The upsurge in cases is thought to be caused by the expanding geographic range of *Ixodes* ticks due to climate change and the increasing human encroachment upon woodland territory [3]. There are currently no available vaccines or therapeutics to treat POWV infection and disease. Thus, avoiding tick-infested areas is the only effective method to prevent infection.

There are two distinct but serologically indistinguishable lineages of POWV: lineage I, prototype POWV, and lineage II, deer tick virus (DTV). While POWV is maintained in an enzootic cycle with ticks (*Ixodes cookei* and *Ixodes marxi*) and medium-sized mammals [4], DTV is maintained by *Ixodes scapularis* and white-footed mice (*Peromyscus leucopus*), but recent evidence also implicates shrews as a potential reservoir [5]. POWV and DTV share 84% nucleotide and 94% amino acid sequence identity [6], and it is thought that DTV diverged approximately 200 years ago [7].

Vaccines under development for POWV have shown significant protection from POWV disease and include: (i) an mRNA vaccine, (ii) a synthetic DNA vaccine delivered by electroporation at the injection site, (iii) a virus-like particle (VLP) vaccine, and (iv) a nanoparticle vaccine bearing the POWV EDIII [8,9,10,11]. Eliciting a robust and protective immune response is essential during vaccine design. The first step in mounting an effective immune response is pathogen recognition by the infected cell. There is increasing evidence that vertebrate RNA viruses subvert host detection through the suppression of the dinucleotide frequency of cytosine followed by guanine (CpG) within their genomic sequences. This suppression prevents recognition and binding by the antiviral host factor zinc-finger antiviral protein (ZAP) [12]. ZAP inhibits viral replication through the recruitment of the RNA degradation machinery and the suppression of virus translation [13,14]. Goncalves-Carneiro et al. recently demonstrated that ZAP-dependent attenuation for enterovirus A71 of the *Picornaviridae* family successfully provided protective immunity in mice [15]. Therefore, artificial increases in CpG dinucleotide frequency, or alternatively UpA (uracil followed by adenine), while preserving amino acid sequence, may have the potential to attenuate POWV by increasing its susceptibility to host immune responses [16].

A live-attenuated vaccine for POWV has not been reported to date but would afford numerous benefits. The use of live-attenuated vaccines mimics natural virus infection, prolongs exposure to viral antigens, and stimulates an immune response similar to natural infection [17]. The yellow fever vaccine (YFV-17D) has proven to be extremely efficacious and provides lifelong immunity to YFV infection [18]. The virulent YFV strain Asibi was passaged in rhesus macaques with intermittent passages in *Aedes aegypti*, followed by serial passages in embryonic mouse and chicken tissue resulting in the YFV vaccine strain 17D [19,20]. The change in virulence of YFV-17D is not yet fully understood and is under active investigation. Protection by YFV-17D is mediated by both B- and T-cell responses, with the envelope protein serving as the immunodominant antigen [21,22,23,24]. The envelope protein of *Flaviviruses* is composed of three separate structural envelope domains (ED): EDI, EDII, and EDIII [25]. The EDIII domain is essential in flavivirus attachment and is a known target of potent flavivirus neutralizing antibodies [26,27,28]. Two licensed human vaccines that use YFV-17D as a vector with its surface antigen swapped for another specific virus include the JEV vaccine (Imojev) and DENV vaccine (Dengvaxia) [29,30]. Another vaccine, similarly constructed using YFV-17D as a backbone and the surface antigen of WNV, is approved for use in horses [31]. We therefore hypothesized that replacing the YFV-17D structural genes (prM-E) with those of POWV may generate an effective vaccine.

We employed two distinct strategies to develop a live-attenuated POWV vaccine. For the first strategy, we recoded the POWV genome to increase its susceptibility to ZAP and the innate immune response. However, attenuation of the recoded viruses was minimal and precluded their use as safe vaccine candidates. Our second approach modified the successful YFV-17D vaccine to express the structural genes prM-E of POWV, and additionally modified NS1 of YFV to lack the first N-linked glycosylation motif. This vaccine candidate significantly protected C57BL/6 mice from lethal POWV challenge. This work establishes a new tool and vaccination strategy to prevent POWV disease in mice that will inform the development of POWV prophylactic treatments in humans.

## 2. Materials and Methods

### 2.1. Cell Culture

Huh-7.5 cells (*H. sapiens*; sex: male) [32], HEK-293T cells, and ZAP knockout (KO) HEK-293T cells [33] (*H. sapiens*; sex: female) were maintained at 37 °C and 5% CO_2_ in Dulbecco’s Modified Eagle Medium (DMEM, Gibco, Grand Island, NY, USA, Thermo Fisher Scientific, Waltham, MA, USA, cat. #11995065) supplemented to contain 0.1 mM nonessential amino acids (NEAA, Gibco, Thermo Fisher Scientific, cat. #11140076) and 10% fetal bovine serum (FBS, HyClone Laboratories, Logan, UT, USA Lot #AUJ35777). All cells tested negative for mycoplasma contamination.

### 2.2. Plasmid Construction

#### 2.2.1. Generation of a Full-Length DTV Infectious Clone

To facilitate sequence engineering and the generation of infectious RNA for virus stock production, a single infectious clone for DTV, designated pACNR-FL-POWV-DTV, was constructed. DTVp1 and DTVp2 [34], kindly provided by Aaron Brault at The Centers for Disease Control and Prevention (CDC), together contain cDNA of the DTV genome (Spooner strain), with DTVp1 containing cDNA encompassing the 5′ end of the genome to an internal BstZ17I site, and DTVp2 containing sequences from the BstZ17I site to the 3′-end of the genome. The single plasmid infectious DTV cDNA clone was produced by cloning the DTV genome from DTVp1 and DTVp2 into the low-copy-number ampicillin-resistant plasmid used to generate the YFV-17D infectious clone (pACNR-2015FLYF-17Da) (GenBank: MT114401.1).

To achieve this, a geneblock (GB), encoding the pACNR vector sequence from a NotI site through the Sp6 promoter sequence followed by the DTV 5′-end to the BamHI site in DTV, was ordered from Integrated DNA Technologies, Inc. (IDT, San Diego, CA, USA) and amplified by PCR using primers RU-O-26915 (F: 5′-TCGACGCGGCCGCTAGCGATG-3′) and RU-O-26916 (R: 5′-CCATAGGATCCCCAGCATGCG-3′), and Phusion High-Fidelity DNA Polymerase (New England Biolabs—NEB, Ipswich, MA, USA, Thermo Fisher Scientific, cat. #F530). The following conditions were used for PCR: initial denaturation at 98 °C for 30 s, 30 cycles of 98 °C for 10 s, 65 °C for 30 s, and 72 °C for 30 s, followed by a final extension for 7 min at 72 °C.

To remove the majority of the YFV genome from pACNR-2015FLYF-17Da but preserve the ampicillin-resistance gene and SP6 promoter, the plasmid was digested with BamHI (NEB), and the backbone segment (6740 bp) was ligated with itself using Ligation High Ver.2 (Toyobo, New York, NY, USA, cat. #LGK-201) to create pACNR-2015FLYF-17Da/Bam. pACNR-2015FLYF-17Da/Bam and the amplified GB were then digested with BamHI and NotI (NEB) and ligated, replacing the 5′-end of the YFV genome with DTV to generate plasmid pACNR-Not-SP6-DTV/Bam. pACNR-Not-SP6-DTV/Bam, and DTVp1 was digested with BamHI and ClaI (NEB) and ligated to replace additional YFV sequences with the BamHI to ClaI region of DTV to create pACNR-Not-SP6-DTV/Cla.

A mutation was introduced in the DTVp2 plasmid to change the NotI runoff site to match the pACNR-2015FLYF-17Da AflII runoff site. PCR using primers RU-O-26318 (F: 5′-CACCTGAAAGGCCAATGTCG-3′) and RU-O-26917 (R: 5′-CCTCGGTTAATTAACTTAAGCGGGTGTTTTTCCGAGTC-3′) was performed with Phusion High-Fidelity DNA Polymerase (Thermo Fisher Scientific, cat. #F530) with the following conditions: initial denaturation at 98 °C for 30 s, 30 cycles of 98 °C for 10 s, 63 °C for 30 s, and 72 °C for 30 s, followed by a final extension for 7 min at 72 °C. RU-O-26917 contains an AflIII site rather than the NotI site, but is otherwise identical with the corresponding sequence within DTVp2. The PCR product and DTVp2 were digested with SalI (NEB) and PacI (NEB) and ligated together to produce DTVp2/AflII-runoff.

Assembly PCR joined the ClaI-BstZ17I region of DTVp1 and the BstZ17I-AflII region of DTVp2/AflII-runoff. DTVp1 was amplified with Phusion High-Fidelity DNA Polymerase (Thermo Fisher Scientific, cat. #F530) using primers RU-O-30341 (F: 5′-TTCACACGGACCAGAGCATGTGGA-3′) and RU-O-30342 (R: 5′-AACAAGCAAACCCAGCATGAGTATACCTCCCCATAAAATAGATGTG-3′) with the following PCR conditions: initial denaturation at 98 °C for 30 s, 30 cycles of 98 °C for 10 s, 69 °C for 30 s, and 72 °C for 15 s, followed by a final extension for 7 min at 72 °C. DTVp2/AflII-runoff was amplified using primers RU-O-30343 (F: 5′-CACATCTATTTTATGGGGAGGTATACTCATGCTGGGTTTGCTTGTT-3′) and RU-O-30344 (R: 5′-CGGAGAACCTGCGTGCAATCCATC-3′) with the following PCR conditions: initial denaturation at 98 °C for 30 s, 30 cycles of 98 °C for 10 s, 69 °C for 30 s and 72 °C for 2 min, followed by a final extension for 7 min at 72 °C. RU-O-30342 and RU-O-30343 are complementary primers that allow for assembly PCR. The PCR products were added together with the following PCR conditions: initial denaturation at 98 °C for 30 s, 30 cycles of 98 °C for 10 s, 69 °C for 30 s and 72 °C for 2 min 15 s, followed by a final extension for 7 min at 72 °C. After 10 cycles, RU-O-30341 and RU-O-30344 were added to the reaction. The blunt end PCR product was TOPO-cloned into the pCR Blunt II-TOPO vector following the Zero Blunt TOPO PCR Cloning Kit (Invitrogen, Carlsbad, CA, USA, Thermo Fisher Scientific, cat. #450245) protocol. The assembly PCR product and pACNR-Not-SP6-DTV/Cla were digested with ClaI and AflII (NEB) and ligated to generate pACNR-FL-POWV-DTV. The construct (GenBank: OQ405350) was confirmed by Sanger sequencing.

#### 2.2.2. Construction of Infectious Clones for Recoded DTV with Increased CpG and UpA Frequencies

Based on the pACNR-FL-POWV-DTV, regions of interest were selected based on availability and access to unique enzyme restrictions. Two ~1.5 kb regions were used for CpG and UpA recoding (Region 1: ClaI to BstEII, Region 2: BstEII to BstAPI). To verify that no critical RNA structures reside in the regions selected, each region was randomized to preserve amino acid structure and dinucleotide frequencies, using the CDLR (C = Codon, D = Dinucleotide, L = Scramble, R = Randomize) algorithm, which uses a complex scrambling method to randomize the order of codons within the sequence while maintaining coding and dinucleotide frequencies through substitutions between equivalently coding triplets in the same upstream and downstream dinucleotide contexts. A resulting CDLR mutant generated by this method has identical coding and dinucleotide frequencies to wildtype (WT) virus [35]. After confirmation that no critical RNA structures reside in these two regions (see Results), four other mutants per region, which include two CpG mutants and two UpA mutants, were designed. A total of ten CpG/UpA recoded regions were synthesized and delivered in ampicillin-resistant plasmids from ThermoFisher Scientific (Waltham, MA, USA), eight being in the pMA-T backbone vector (R1_CDLR, R2_CDLR, R1-CpG-1, R1-CpG-2, R2-CpG-1, R2-CpG-2, R1-UpA-1 and R1-UpA-2), and two being in pMA-RQ (R2-UpA-1 and R2-UpA-2). Sequences of the recoded regions can be found in Supplementary File S1. Plasmids and pACNR-FL-POWV-DTV were digested with the respective enzymes and the appropriate pieces ligated using Ligation High Ver.2 (Toyobo, cat. #LGK-201). All constructs (GenBank: OQ405351, OQ405352, OQ405353, OQ405354, OQ405355, OQ405356, OQ405357, OQ405358, OQ405359, and OQ405360) were confirmed by Sanger sequencing. A schematic of the recoded DTVs can be found in Figure S1.

#### 2.2.3. Generation of Three Chimeric YFV-17D-DTV Infectious Clones

Chimeric viruses containing prM-E of DTV in the backbone of YFV-17D were produced by replacing the YFV genes in pACNR-2015FLYF-17Da with homologous DTV genes. Three gene fragments (GB2a: YFV-DTV-prM-E, GB2b: YFV-DTV-Sig-prM-E, and GB3: DTV E-YFV NS1) were ordered from IDT. Gene fragments GB2a and GB2b contained sequences upstream of the NotI site in front of YFV in pACNR-2015FLYF-17Da, the YFV 5′-UTR and capsid (C) fused to DTV prM, and the 5′ region of DTV E. GB 2a and 2b differ in the viral signal sequence (Sig) upstream of the prM sequence, but are otherwise identical; 2a contains the YFV signal sequence while 2b contains the DTV signal sequence. GB3 encoded the 3′ region of DTV E fused to YFV NS1-encoding sequences, extending beyond a unique MluI site. The sequences of GB2a, GB2b, and GB3 can be found in Supplementary File S1. Fragments GB2a and GB2b were PCR amplified using Phusion High-Fidelity DNA Polymerase (Thermo Fisher Scientific, cat. #F530) with primers RU-O-29343 (F: 5′-GTCGACGCGGCCGCTAGCGATGAC-3′) and RU-O-29344 (R: 5′-TGAGTCATAAACATGACCTACAGC-3′), while GB3 was PCR amplified with primers RU-O-29345 (F: 5′-GCCTGTGCCAAGTTTGAATG-3′) and RU-O-29346 (R: 5′-CATGTACACGCGTGTGGTGAACAC-3′) under the following conditions: initial denaturation at 98 °C for 30 s, 30 cycles of 98 °C for 10 s, 63 °C for 30 s, and 72 °C for 1 min, followed by a final extension for 7 min at 72 °C. GB2a and GB3 were combined by assembly PCR using the following conditions: initial denaturation at 98 °C for 30 s, 30 cycles of 98 °C for 10 s, 69 °C for 30 s and 72 °C for 1 min, followed by a final extension for 7 min at 72 °C, with the addition of primers RU-O-29343 and RU-O-29346 after 10 cycles. GB2b and GB3 were combined under identical conditions. The blunt end PCR products were TOPO-cloned into the pCR Blunt II-TOPO vector following the Zero Blunt TOPO PCR Cloning Kit (Thermo Fisher Scientific, cat. #450245) protocol. The assembly PCR products contained in the TOPO clones and pACNR-2015FLYF-17Da were each digested with NotI (NEB) and MluI (NEB), and the appropriate fragments were ligated together using Ligation High Ver.2 (Toyobo, cat. #LGK-201) to generate Chimera I (YFV-17D with DTV prM-E) and Chimera II (YFV-17D with DTV Sig-prM-E). The constructs (GenBank: OQ405361 and OQ405362) were confirmed by Sanger sequencing. A schematic of the chimeric viruses can be found in Figure S1.

##### Generation of Chimera I Containing a Mutation of the YFV NS1 N-Linked Glycosylation Site (CIGM)

Chimera I was amplified by PCR using Phusion High-Fidelity DNA Polymerase (Thermo Fisher Scientific, cat. #F530) with primers RU-O-29343 (5′-GTCGACGCGGCCGCTAGCGATGAC-3′) and RU-O-31248 (5′-CTATGATGAAGCTTCCTTGCTTCCTCCCT-3′) under the following conditions: initial denaturation at 98 °C for 30 s, 30 cycles of 98 °C for 10 s, 64 °C for 30 s, and 72 °C for 1 min 40 s, followed by a final extension for 7 min at 72 °C. Chimera I was amplified again with a second set of primers, RU-O-31249 (5′-AGGGAGGAAGCAAGGAAGCTTCATCATAG-3′) and RU-O-31250 (5′-CCAAGATAGATCCATCGCAGTC-3′), under the following conditions: initial denaturation at 98 °C for 30 s, 30 cycles of 98 °C for 10 s, 58 °C for 30 s, and 72 °C for 6 s, followed by a final extension for 7 min at 72 °C. RU-O-31248 and RU-O-31249 are complementary and allow for assembly PCR, introducing two point mutations to mutate the asparagine to glutamine within the N-linked glycosylation motif (Asn-Gly-Ser). Assembly PCR combining the two PCR products was performed under the following conditions: initial denaturation at 98 °C for 30 s, 30 cycles of 98 °C for 10 s, 61 °C for 30 s, and 72 °C for 1 min 40 s, followed by a final extension for 7 min at 72 °C. After the first 10 cycles, RU-O-29343 and RU-O-31250 were added to the reaction. The blunt end PCR product was TOPO-cloned into the pCR Blunt II-TOPO vector following the Zero Blunt TOPO PCR Cloning Kit (Fisher Scientific, cat. #450245) protocol. The assembly PCR product in the TOPO clone and Chimera I were both digested with MluI (NEB) and NotI (NEB) and ligated together using Ligation High Ver.2 (Toyobo, cat. #LGK-201) to generate Chimera I Glycosylation Mutant (CIGM). The construct (GenBank: OQ405363) was confirmed by Sanger sequencing. A schematic of CIGM can be found in Figure S1.

#### 2.2.4. Generation of Plasmid Used for POWV RNA Standard

DTVp2 and pcDNA3.1^+^ plasmid were digested with KpnI (NEB) and NotI (NEB) and ligated to create pcDNA3.1-POWV-DTV(4986-10837).

### 2.3. Virus Production and Titration

WT DTV was generated by digesting DTVp1 and DTVp2 with BstZ17I (NEB). The two linearized plasmids were ligated in vitro, and the product was digested with NotI. The standard AmpliScribe T7-Flash Transcription Reaction (Epicentre Biotechnologies, Madison, WI, USA) protocol was followed with the following modifications: omission of RiboGuard RNase Inhibitor, the addition of Cap Analog (NEB, cat. #S1407) with a final concentration of 1.8 mM, DTT at 9 mM, ATP at 1.4 mM, and GTP, UTP, and CTP all at 6.8 mM.

All pACNR cDNA infectious clones (pACNR-2015FLYF-17Da, pACNR-FL-POWV-DTV, CpG/UpA recoded mutants, Chimera I, Chimera II, and Chimera I Glycosylation Mutant) contain the same upstream SP6 promoter sequence and AflII runoff site. The DNA template was digested with AflII, cleaned with phenol-chloroform-isoamyl alcohol, and ethanol precipitated. RNA was synthesized from 1–2 μg linear DNA template using the mMessage mMachine SP6 Transcription Kit (Ambion, Austin, TX, USA, Thermo Fisher Scientific, cat. #AM1340) according to the manufacture’s protocol. TURBO DNase (Ambion, Thermo Fisher Scientific, cat. #AM2238) was added after the reaction and the RNeasy Minikit (Qiagen, Redwood City, CA, USA, cat. #74104) was used to clean up the RNA. RNA was quantified using a NanoDrop ND-1000 spectrophotometer and stored in 5 μg aliquots at −80 °C.

To generate virus stocks, 5 μg of in vitro transcribed viral RNA was electroporated using a BTX Electro Square Porator ECM 830 at 3.8 kV/cm field strength into 6 × 10^6^ Huh-7.5 cells diluted into 400 µL ice-cold PBS using 2-mm gap electroporation cuvettes. Cells of three individual electroporation reactions were combined and seeded into a poly-L-lysine coated T-175 flask. Media was replaced after 4–6 h to remove excess RNA and non-attached cells.

Supernatants were harvested at one day post-infection (dpi) (YFV-17D, Chimera I, Chimera II, Chimera I Glycosylation Mutant), 2 dpi (pACNR-FL-POWV-DTV, pACNR-FL-POWV-DTV-[R1_CDLR, R2_CDLR, R1-CpG-1, R1-UpA-1, R1-UpA-2, R2-CpG-1, R2-CpG-2, R2-UpA-1, R2-UpA-2]), 6 dpi (pACNR-FL-POWV-DTV-R1-CpG-2), or 7 dpi (DTV from DTVp1 and DTVp2), clarified using an Allegra X-12R centrifuge (Beckman Coulter, Indianapolis, IN, USA) at 2095× *g* for 10 min at 4 °C, and stored in 1.8 mL aliquots at −80 °C. Aliquots were subsequently thawed and further aliquoted for single use. Titers were measured via standard plaque assay (see below) using aliquots that had undergone two freeze/thaw cycles to mimic the viral stock that would be injected into mice. All experiments with virus were performed using biosafety level (BSL)3 conditions.

Virus titers were quantified using standard plaque assays on Huh-7.5 cells. Cells seeded into six-well plates (4 × 10^5^ per well) were incubated for 24 h and then washed with 2 mL of Opti-MEM. Cells were then infected for 1 h with 500 μL of serial 10-fold virus dilutions in Opti-MEM. The virus inoculum was removed, and a semisolid overlay consisting of DMEM containing 10% FBS and 1.2% microcrystalline cellulose (Avicel, FMC Biopolymer, Philadelphia, PA, USA) was added to the cells. After incubation for 4 d (YFV-17D, Chimera I, Chimera II), or 6 d (DTV from DTVp1 and DTVp2, Chimera I Glycosylation Mutant, and all CpG and UpA recoded DTV), cells were fixed using 7% formaldehyde and stained with crystal violet for plaque enumeration.

### 2.4. POWV EDIII Expression and Purification

DTV EDIII was generated by recombinant expression in *Escherichia coli* and purified from inclusion bodies under denaturing conditions as previously described [36,37,38] with minor modifications. The plasmid construct, generously provided by Jennifer Keefe and Pamela Bjorkman (Caltech), contained codon optimized EDIII encoding sequences in the pET21 plasmid backbone. Briefly, expression was induced with isopropyl-β-D-thiogalactopyranoside at log phase for 4 h and the bacteria were harvested by centrifugation. After lysis using an ultrasonic cell disruptor (Heat Systems Ultrasonic Convertor CL4) and centrifugal clarification, the pellet was resuspended in Tris-buffered 6 M guanidine hydrochloride and clarified again. After the addition of 20 mM β-mercaptoethanol, EDIII in the supernatant was refolded by dropwise, rapid dilution at 4 °C into 100 mM Tris-base pH 8.0 containing 400 mM L-arginine, 2 mM EDTA, 5 mM reduced glutathione, 0.5 mM oxidized glutathione, and 10% glycerol. After concentration using a stirred ultrafiltration cell (Fisher Scientific, cat. #5123), the protein was purified by size exclusion chromatography with the HiLoad 16/600 Superdex 200 column (Cytiva, Marlborough, MA, USA, cat. #28989335) in 20 mM Tris pH 8.0, 0.5 M NaCl, and was stored at 4 °C. Prior to use as an immunogen, endotoxin was removed using the ToxinEraser^TM^ Endotoxin Removal Kit (Genscript, Piscataway, NJ, USA, cat. #L00338), and the endotoxin levels were measured using the ToxinSensor^TM^ Endotoxin Detection System (Genscript, cat. #L00350), following the manufacturer’s instructions.

To inject into C57BL/6 (BL6) mice, 25 ng of protein was mixed at a 1:1 volume of either Freund’s Incomplete Adjuvant or Magic Mouse Adjuvant. The mixture was vortexed for ~10 min until a thick emulsion developed, and the mixture in its entirety (100 μL) was then administered to one mouse by the subcutaneous (s.c.) route.

### 2.5. Vaccination Schemes

**Figure 1:**
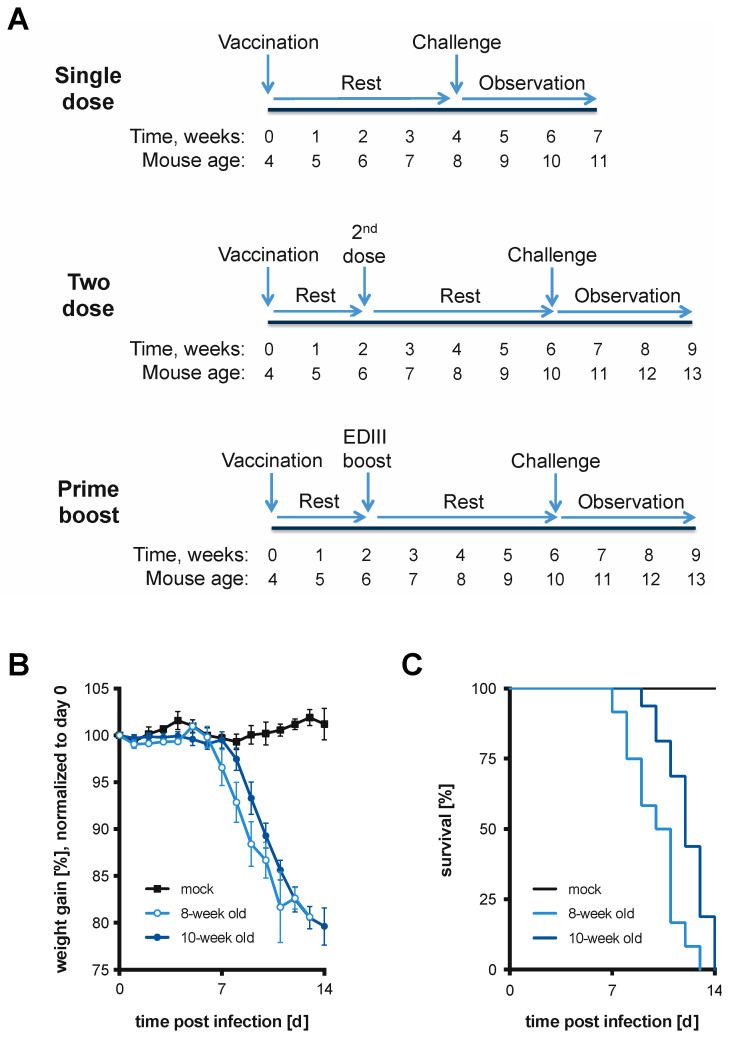
Vaccinations schemes and murine model of Powassan virus disease. (**A**) Depiction of vaccination schemes that were used to test vaccine efficacy. Four-week-old BL6 mice were vaccinated and (i) challenged with a lethal dose of DTV four weeks later (Single dose). In two additional vaccination regimens, two weeks after the first vaccination mice received (ii) a second live-attenuated virus vaccination (Two dose) or (iii) a protein boost with DTV EDIII (Prime boost), followed by DTV challenge four weeks later. Upon challenge, mice were observed for weight loss and disease signs for up to three weeks. (**B**) Mice infected with 2.5 × 10^4^ PFU of DTV via the s.c. route were monitored daily for weight loss and disease signs and sacrificed upon meeting humane endpoints. The average normalized weight of infected eight-week-old (*n* = 12) and 10-week-old (*n* = 16) mice is plotted. Weights were normalized to the day of inoculation (day 0). Error bars indicate standard deviation (SD). (**C**) Survival curves of mice challenged with DTV in (**B**) are plotted.

Three vaccinations schemes (Figure 1A) were designed to test the vaccine candidates: (i) single-dose, (ii) homologous two-dose, and (iii) heterologous two-dose using the EDIII protein as a boost. The first dose was administered to four-week-old BL6 mice, and the second doses were administered two weeks later. Immunizations were administered s.c. at the rear end of the mouse, near the tail. In a small number of experiments, the intraperitoneal (i.p.) route was utilized. Following the final injection, mice were allowed to rest for four weeks before s.c. challenge with a lethal dose (2.5 × 10^4^ plaque-forming units [PFU]) of WT DTV. Mice were then observed for another three weeks for signs of POWV disease and weight loss. The age of the mice at the time of lethal challenge was eight weeks (single-dose regimen) or 10 weeks (two-dose or prime-boost regimens). Because the disease process was under study, analgesics were not administered, but humane endpoints, including the loss of >20% of peak weight or the inability to ambulate, were used; euthanized animals were considered to have succumbed to the infection.

### 2.6. Mouse Infections and Monitoring/Scoring

All experiments utilized BL6 mice, of both sexes, bred and housed at the American Association for Accreditation of Laboratory Animal Care (AAALAC)-accredited Rockefeller University Comparative Biosciences Center (CBC). All experimental procedures involving DTV infection were conducted under BSL3 containment using protocols reviewed and approved by both the Rockefeller University’s Institutional Animal Care and Use Committee (IACUC protocol number 22004-H) and the Department of Defense Animal Care and Use Review Office (ACURO protocol TB180020.e001).

The health of infected mice was monitored based on their daily weight and a scoring system. Mice that reached humane endpoints during this monitoring interval were humanely euthanized using the Euthanex Smartbox System (CO_2_). Cervical dislocation was performed as a secondary method of euthanasia. A score of “0” was given to healthy mice, “1” for ruffled fur/hunched back, “2” for weakness in limbs, “3” for tremors/shaking, “4” for paralysis, and “5” for death. Weakness in limbs was determined by observing the mouse’s ability to pull itself up from the edge of the cage. Significant tremors/shaking due to weakness were determined by lifting the mouse up by its tail. Paralysis was frequently seen in the hind limbs of the mice but was also occasionally observed in the front limbs.

Mice were immobilized using isoflurane anesthesia prior to s.c. injection of 200 μL virus diluted in PBS or 100 μL of EDIII protein diluted in adjuvant.

### 2.7. Tissue Harvest, Virus Inactivation, and RNA Extraction

For harvesting of tissues from vaccinated or infected mice, animals were euthanized, and blood was collected by cardiac puncture with a 29-gauge needle. Serum was obtained by centrifugation of blood at 20,800× *g* for 5 min. Heart, liver, spleen, kidney, and brain were resected, divided evenly into tubes containing phosphate-buffered saline (PBS) or TRIzol reagent (Invitrogen, Carlsbad, CA, USA), and homogenized using a MagNA lyser (Roche) at 6000 rpm for 60 s with 1-mm diameter glass beads (BioSpec Products, Bartlesville, OK, USA, cat. #11079110).

RNA was isolated from TRIzol-inactivated tissues and serum by chloroform extraction, ethanol precipitation, and purification using an RNeasy kit (Qiagen, cat. #74104).

### 2.8. Generation of RNA for Use as RT-qPCR Standards

To generate an RNA standard for POWV NS5 quantification, pcDNA3.1-POWV-DTV(4986-10837) was digested with NotI (NEB). The T7 RiboMAX^TM^ Express Large Scale RNA Production system (Promega, Madison, WI, USA, cat. #P1320) was used to generate RNA following the manufacturer’s instructions. RNA cleanup was performed using an RNeasy kit (Qiagen, Redwood City, CA, USA, cat. #74104) following the manufacturer’s protocol. To generate an RNA standard for YFV-17D NS5 quantification, the YFV-17D infectious clone was linearized and genomic RNA was transcribed and purified by lithium chloride precipitation using the mMessage mMachine SP6 Transcription kit (Ambion, Austin, TX, USA, Thermo Fisher Scientific, cat. #AM1340), following the manufacturer’s protocol.

### 2.9. Quantification of Genome Copies by RT-qPCR for YFV and DTV

Genome copies of YFV and DTV were quantified by one-step reverse transcription-quantitative PCR (RT-qPCR) using the Roche LightCycler 480 RNA Master Hydrolysis Probe kit (Roche, cat. #04991885001), following the manufacturer’s protocol. YFV- and DTV NS5 sequence-specific primers (YFV F: 5′-GCTGTTTCCTCAGCTGTTCC-3′ and R: 5′-GTCTTCCGTGGTCATCCACT-3′; DTV F: 5′-TGCCTGTCCAAGGCGTAC-3′ and R: 5′-ATATGGCGAAACCAAGGGTGC-3′) were used with an internal fluorogenic probe (YFV probe: 5′-6-carboxyfluorescein [FAM]-ATGGTCGAT-ZEN-TCATGGGAAAG-/Iowa Black FQ quencher [IABkFQ]-3′; DTV probe: 5′-6-FAM-GGCAGATGT-ZEN-GGCTTCTCAA-IABkFQ-3′). To relate threshold cycle (C_T_) values to copies of genomic RNA, standard curves were generated from 10-fold dilutions, from 10^1^–10^8^ copies, of in vitro-transcribed genomic YFV-17D RNA or NS5-containing DTV RNA (see above).

All qPCRs were performed in three technical replicates. Samples with no detection of YFV or DTV by qPCR were given an artificial value of 1, a value lower than the limit of detection (LOD) of 25 genome equivalents per µg RNA.

### 2.10. Sequence Analysis of Viral RNA Isolated from Brain

To determine the sequence of purified viral RNA isolated from brain tissue, viral RNA was reverse transcribed into cDNA using SuperScript^TM^ III First-Strand Synthesis System (Invitrogen, Carlsbad, CA, USA, Thermo Fisher Scientific, cat. #18080051), according to the manufacturer’s protocol, using random hexamers as primers. The cDNA was amplified using two sets of POWV-specific primers (5′-GGTCGTAAAGCTCAGGGAAA-3′ and 5′-ACATAGCTGAAATGGGAGCC-3′; 5′-CAGGAACGGAGAGGTGATTG-3′ and 5′-GTATTCTGTGGATGCTGACCTG-3′) and Phusion High-Fidelity DNA Polymerase (Thermo Fisher Scientific, cat. #F530). The following conditions were used for PCR: initial denaturation at 98 °C for 30 s, 30 cycles of 98 °C for 10 s, 63 °C for 30 s, and 72 °C for 3 min, followed by a final extension for 7 min at 72 °C. cDNA was submitted to the Center for Computational and Integrative Biology (CCIB) DNA Core Facility, Massachusetts General Hospital (Cambridge, MA, USA) for high throughput amplicon sequencing.

## 3. Results

### 3.1. Lethal Model of DTV Infection in 8- and 10-Week-Old BL6 Mice

A lethal murine model of DTV infection in BL6 mice was established to test the efficacy of live-attenuated vaccine candidates in multiple vaccination schemes (Figure 1A). Based on our planned vaccination regimens, mice were challenged at eight or 10 weeks of age. Infection via the subcutaneous route (s.c.) was chosen as it more closely mimics a natural infection in which the virus is transmitted by a tick bite. Published studies of DTV infection via s.c. inoculation in BL6 mice showed 57% and 60% lethality in seven- and 14-week-old mice using 10^2^ focus forming units (FFU) of DTV, Spooner strain [8]. In our hands, a s.c. dose of 2.5 × 10^4^ PFU DTV resulted in 100% mortality in both eight- and 10-week-old mice after 14 days (Figure 1B,C). A dose of 10^4^ PFU DTV resulted in 75–80% mortality. At seven days post-infection, DTV-infected mice exhibited disease signs including ruffled fur, a hunched back, lethargy, and paralysis (primarily in hind limbs). Dramatic weight loss was also observed starting at the onset of these disease signs.

### 3.2. CpG and UpA Recoded DTV Viruses

**Figure 2:**
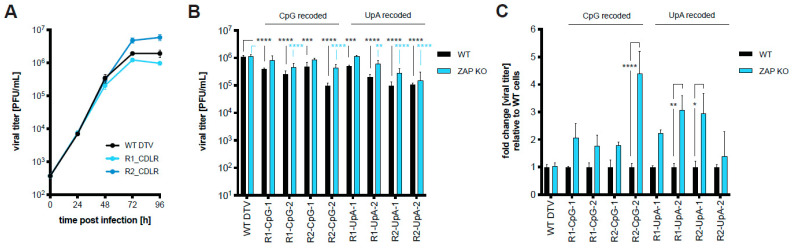
Replication kinetics of CpG- and UpA-recoded DTV constructs. (**A**) Huh-7.5 cells were infected with WT DTV and CDLR recoded control viruses (MOI = 0.01 PFU/cell). Supernatants were harvested daily for 4 d and infectious virus was titered by plaque assay. (**B**) WT and ZAP KO HEK-293T cells were infected (MOI = 1 PFU/cell) with WT and CpG or UpA recoded DTV. Supernatants were collected after 24 h and infectious virus was titered by plaque assay. **, *p* < 0.005; ***, *p* < 0.0005; ****, *p* < 0.0001, two-way ANOVA with Dunnett’s multiple comparison test. (**C**) For each virus, titers from (**B**) were normalized to that of the WT HEK-293T cells to indicate the fold change in virus titers produced in ZAP KO compared to WT cells. *, *p* < 0.05; **, *p* < 0.01; ****, *p* < 0.0001, two-way ANOVA with Šidák’s multiple comparisons test. (**A**–**C**) Mean titers of triplicate samples are plotted; error bars indicate the SD.

Our initial strategy for generating live-attenuated POWV vaccine candidates was to recode the DTV genome to increase the content of CpG or UpA dinucleotides while maintaining the protein sequence. Huh-7.5 cells, which are permissive for WT DTV infection, were infected at a low MOI (0.01 PFU/cell) with WT DTV and DTV controls (R1_CDLR and R2_CDLR), which contain identical dinucleotide frequencies, scrambled within region 1 (NS1 to NS3-encoding) or region 2 (NS3-encoding) to assess whether important secondary RNA structures reside in those regions, which would preclude their use for recoding. Supernatants were harvested at 24, 48, 72, and 96 h post-infection (hpi), and virus production was quantified by plaque assay (Figure 2A). The recoded control viruses were viable and replicated to similar levels as WT DTV, indicating that no essential RNA structures reside in these regions. Based on these results, viruses were generated in which the genomic regions “R1” and “R2” were recoded for increased CpG or UpA frequencies (Table 1). To determine whether this change in dinucleotide frequency affects replication in vitro in a ZAP-dependent manner, WT HEK-293T and ZAP KO cells were infected with WT DTV as well as CpG- and UpA-recoded viruses at two different MOI (1 and 0.1 PFU/cell) to assess initial infection at an early time point and viral spread over time. These cells were chosen as we have documented that the intracellular milieu supports ZAP’s antiviral function [39]. The replication of the recoded viruses in WT cells infected at the higher MOI was reduced for each of the mutants compared to WT DTV (Figure 2B). When comparing the replication of the CpG- and UpA-recoded viruses in WT compared to ZAP KO cells, we found moderate (~2–4 fold) increases in ZAP KO cells which were statistically significant for R2-CpG-2, R1-UpA-2, and R2-UpA-1 (Figure 2C). However, these differences were not consistently observed when infecting at the lower MOI and measuring virion output across the time course of the growth curve (Figure S2).

To determine whether these recoded viruses were attenuated in vivo, and thus suitable for use as vaccine candidates, four-week-old BL6 mice (*n* = 5) were infected with 10^3^ PFU and monitored for clinical signs of disease and weight loss. This dose was chosen for the vaccination dose, since younger mice are more susceptible to viral infection, and our intent was to trigger a protective immune response before exposing them to the higher challenge dose of WT DTV. All mice infected with the recoded viruses, except one mouse infected with R1-UpA-1, succumbed to infection with similar kinetics as mice infected with WT DTV, indicating that our recoding strategy to increase CpG and UpA in these regions did not result in significant attenuation in vivo (Figure S3).

### 3.3. Chimeric YFV-17D-DTV Protects against DTV In Vivo

**Figure 3:**
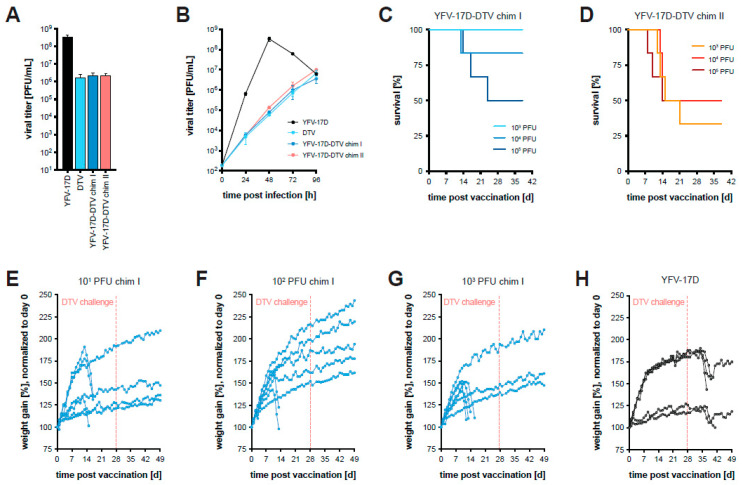
Replication kinetics of chimeric and parental viruses in vitro and in vivo. (**A**,**B**) Huh-7.5 cells were infected with the parental YFV-17D and DTV, and the chimeric viruses I and II at (**A**) MOI = 1 PFU/cell and (**B**) MOI = 0.005 PFU/cell. Supernatants were harvested at the indicated time points and the infectious virus was titered by plaque assay. Mean titers of triplicate samples are plotted; error bars indicate the SD. (**C**,**D**) Survival curves of BL6 mice inoculated i.p. with the indicated doses of Chimera I and II (*n* = 6 per cohort). (**E**–**G**) Normalized weights of BL6 mice vaccinated s.c. with the indicated doses of Chimera I (*n* = 7 per cohort) and challenged with a lethal dose of WT DTV at 28 days post-vaccination. (**H**) Control cohort for (**E**–**G**) where mice were “vaccinated” with 10^3^ PFU of the parental YFV-17D (*n* = 5).

As another strategy, we utilized the highly attenuated YFV-17D vaccine strain [40] to express the DTV structural proteins (prM-E) exposed on the virion surface [41]. The prM and E genes of the YFV-17D vaccine strain were replaced with those of DTV to generate “Chimera I” and “Chimera II”, which differ exclusively in the signal sequence upstream of prM. Chimera I contains the YFV signal sequence and Chimera II contains the DTV signal sequence. To assess viral fitness, Huh-7.5 cells were infected at two different MOI (1 and 0.005 PFU/cell) with the parental and chimeric viruses (Figure 3A,B). The supernatants of the infected cells were harvested at various time points, and the virus was quantified by plaque assay. The replication of both chimeric viruses appeared to be attenuated relative to the parental YFV-17D; however, the replication was nearly identical to that of DTV. YFV-17D is fairly cytopathic and forms large, homogenous clear plaques, while DTV is less cytopathic, and its plaques are rather small and heterogenous. Interestingly, plaques produced on Huh-7.5 monolayers by both chimeric viruses were similar in clarity to those of YFV-17D and distinct from the DTV plaques. Examples of the YFV, DTV, and Chimera I plaque phenotypes are shown in Figure S4.

Given that the infection of BL6 mice with YFV-17D does not result in disease [42], we expected that the YFV-17D-DTV chimeric viruses would show similar attenuation in mice as YFV-17D. As an initial test to determine if the YFV-17D-DTV chimeric viruses maintained the attenuated phenotype, increasing doses (10^3^, 10^4^, 10^5^ PFU) of each chimeric virus were administered by the i.p. route to four-week-old BL6 mice (*n* = 6). Somewhat surprisingly, mortality was observed for Chimera I, in a dose-dependent manner (Figure 3C). All animals survived without showing weight loss when infected with 10^3^ PFU. However, 1/6 and 3/6 mice succumbed to infection with 10^4^ PFU and 10^5^ PFU, respectively. Mice infected with Chimera II succumbed at all three doses at different frequencies without dose dependency (Figure 3D).

To potentially limit the virulence observed during infection by Chimera I virus, four-week-old BL6 mice (*n* = 7) were inoculated with lower doses (10^1^, 10^2^, 10^3^ PFU) via the s.c. route as a more natural route for vaccination (Figure 3E–G). YFV-17D was used as a control at a dose of 10^3^ PFU to match the highest dose of Chimera I (Figure 3H). Chimera I caused mortality in some mice, but those that survived exhibited no signs of disease or even weight loss. The dose of 10^3^ PFU Chimera I resulted in the survival of 3/7, or 43%, of the mice in this experiment. The difference in the survival rate after vaccination with 10^3^ PFU Chimera I (Figure 3C versus Figure 3G) is likely due to the small cohort sizes as well as the vaccination route. Surviving mice, together with YFV-17D infected mice, were challenged four weeks after vaccination with a lethal dose of DTV. All mice vaccinated with Chimera I survived while all mice “mock-vaccinated” with YFV-17D lost weight with clear signs of disease, resulting in mortality in 3/5 mice. These results suggests that a chimeric YFV-17D-DTV expressing the structural proteins prM-E of DTV can provide protection against lethal challenge with DTV in mice, but that the two chimeric viruses were too virulent for consideration as vaccine candidates.

### 3.4. Removal of a Glycosylation Motif within YFV NS1 Further Attenuates Chimera I In Vitro

We were interested in further attenuating Chimera I while preserving its protective phenotype. The removal of glycosylation sites within the E or NS1 region has been shown to reduce virulence for related flaviviruses [43,44,45,46]. In YFV-17D, a mutation removing the first glycosylation site in NS1 reduces both viral replication in cell culture and mouse neurovirulence after intracerebral inoculation [47]. N-linked glycosylation sites were identified through the Asn-X-Ser/Thr consensus sequence where X is any amino acid except proline. We targeted the first N-linked glycosylation site in the YFV NS1 region within Chimera I by site-directed mutagenesis to replace the asparagine (Asn) with glutamine (Gln), to abolish glycosylation at this site while conserving the polar uncharged side chain. We named the virus “Chimera I Glycosylation Mutant” (CIGM).

**Figure 4:**
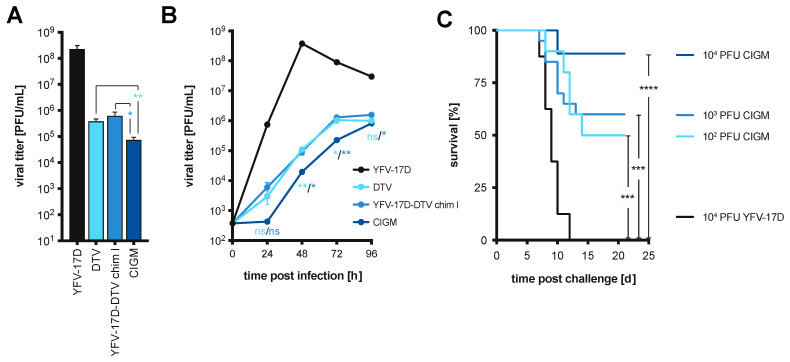
In vitro and in vivo replication kinetics of Chimera I harboring an NS1 glycosylation site mutation. (**A**,**B**) Huh-7.5 cells were infected with YFV-17D, DTV, Chimera I, or Chimera I harboring an NS1 glycosylation site mutation (CIGM), using (**A**) MOI = 1 PFU/cell or (**B**) MOI = 0.01 PFU/cell. Supernatants were harvested at the indicated times and the infectious virus was titered by plaque assay. Statistical significance was determined by Students *t*-test. Light blue, CIGM versus the parental WT DTV; blue, CIGM versus Chimera I. ns, not significant; *, *p* < 0.05, **, *p* < 0.01. (**C**) Survival curves of BL6 mice vaccinated with 10^2^ PFU (*n* = 10), 10^3^ PFU (*n* = 20), and 10^4^ PFU (*n* = 9) CIGM followed by a lethal DTV challenge at 28 days post-vaccination. YFV-17D was used as a control vaccine at 10^4^ PFU (*n* = 8). Statistical significance was determined by the Log-rank (Mantel-Cox) test. ns, not significant; *, *p* < 0.05; **, *p* < 0.01; ***, *p*<0.001; ****, *p* < 0.0001.

Next, we compared the viral replication of CIGM to the parental Chimera I, YFV-17D, and DTV. Similar to previous experiments, Huh-7.5 cells were infected at two different MOI (1 and 0.01 PFU/cell), and supernatants of infected cells were harvested over time for the quantification of virion production by plaque assay (Figure 4A,B). Infection with CIGM was significantly impaired at both MOI relative to Chimera I, and DTV, and markedly impaired compared to YFV-17D. This suggests that the removal of the glycosylation site within YFV NS1 attenuates the YFV-17D-DTV chimeric virus in vitro.

### 3.5. Chimera I Glycosylation Mutant Partially Protects In Vivo against Lethal DTV Challenge

To test whether the CIGM virus retains the protective phenotype observed with Chimera I, CIGM was administered s.c. to four-week-old BL6 mice at increasing doses (10^2^, 10^3^, 10^4^ PFU). YFV-17D was used as a control vaccine at 10^4^ PFU, the highest dose tested for CIGM. Mice were scored, based on observed clinical signs, after vaccination to assess the level of attenuation of the vaccine, as well as after challenge with a lethal dose of DTV four weeks later (Figure S5). One out of nine mice vaccinated with 10^4^ PFU of YFV-17D, as well as 1/10 mice that received 10^4^ PFU of CIGM, displayed disease signs and succumbed after the vaccination. No mice vaccinated with the lower doses of CIGM exhibited any disease signs. After lethal challenge, mice exhibited signs of disease and succumbed to infection in a dose-dependent manner: 90% of mice vaccinated with 10^2^ PFU of CIGM exhibited signs of disease with a 50% survival rate (*n* = 10), 50% of mice vaccinated with 10^3^ PFU of CIGM became sick, with a 60% survival rate (*n* = 20), and 22% of mice vaccinated with 10^4^ PFU of CIGM became sick upon challenge with an 89% survival rate (*n* = 9) (Figure 4C). In contrast, all mice vaccinated with 10^4^ PFU of YFV-17D as a control exhibited signs of disease and succumbed to the DTV challenge (*n* = 8, Figure 4C and Figure S5A). These data indicate that increasing the CIGM dose used to vaccinate the mice improves the chance of survival when challenged with DTV, but that high doses of CIGM are not without serious side effects in some animals.

As mentioned above, 1/10 mice vaccinated with 10^4^ PFU of CIGM succumbed to vaccination on day 19, prior to lethal challenge with DTV (Figure S5D). The animal succumbed to infection with the chimeric virus, as its presence was detected in the brain by RT-qPCR using primers targeting the YFV-17D NS5 region but not with primers targeting the DTV NS5 region. To determine whether reversion of the introduced glycosylation mutation accounts for the observed virulence, RNA was isolated from the brain and subjected to sequencing. There was no indication of reversion of the introduced mutation. The sequencing of this sample indicated a single nucleotide mutation (c.1008A > T) resulting in an amino acid change in the E protein (p.336E > D). We also observed another instance in which a mouse vaccinated with 10^4^ PFU of YFV-17D died on day 24 (Figure S5A), also prior to DTV challenge. It is therefore unclear whether the death of the animal vaccinated with 10^4^ PFU of CIGM is related to the structural genes of DTV, or the YFV-17D backbone, and the role of the E protein mutation is unknown. To avoid further complications, we used 10^3^ PFU of CIGM for future in vivo experiments.

To determine whether the replication of CIGM in specific tissues might contribute to its protective effects, mice were vaccinated with 10^4^ PFU, and animals were sacrificed 2, 4, 6, and 8 dpi (*n* = 3 for each timepoint) to quantify CIGM genome levels. RT-qPCR to detect YFV NS5 was performed on serum and mouse tissues, including the liver, spleen, brain, heart, and kidney. The vaccine candidate was not consistently detected in any tissue. Given the sensitivity of our assay (10 genome equivalents per μg RNA), we suspect that the levels of replication are very low or that the virus may not reach sufficiently high titers to allow hematogenous seeding of the organs. These results suggest that high levels of CIGM replication are not necessary to elicit the immunity required for protection from lethal DTV challenge.

### 3.6. Chimera I Glycosylation Mutant Tested in Two-Dose and Prime-Boost Regimens

**Figure 5:**
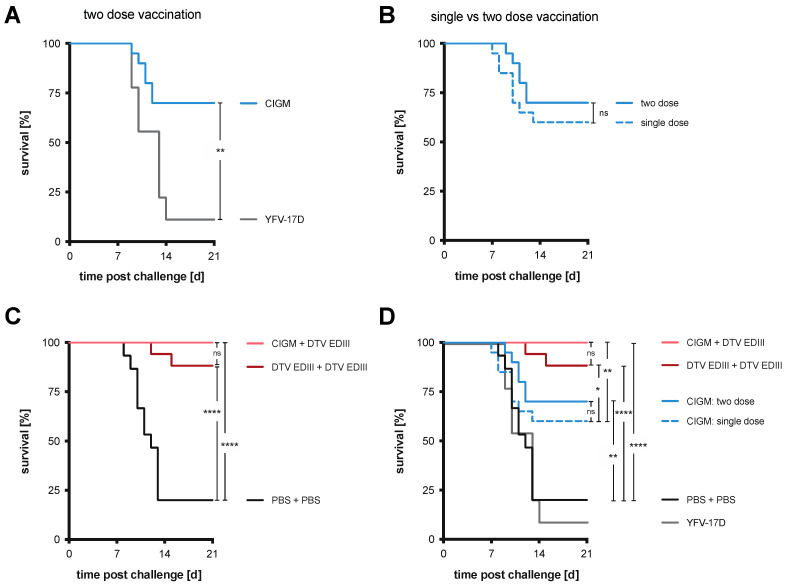
In vivo efficacy of CIGM in different vaccination regimens. (**A**) Survival curves of BL6 mice challenged with a lethal dose of WT DTV after vaccination with two doses of 10^3^ PFU CIGM (*n* = 20). YFV-17D was used in a control cohort (*n* = 9) at 10^3^ PFU. (**B**) Survival curves of BL6 mice challenged with a lethal dose of WT DTV upon vaccination with 10^3^ PFU CIGM comparing a single-dose (*n* = 20) versus the two-dose (*n* = 20) regimen shown in (**A**). Single-dose data is from Figure 4C. (**C**) Survival curves of vaccinated BL6 mice challenged with a lethal dose of WT DTV. Mice were initially vaccinated as indicated with 10^3^ PFU CIGM (*n* = 19) or 25 ng DTV EDIII (*n* = 17), followed two weeks later by a 25 ng dose of DTV EDIII. In a control cohort (*n* = 15), mice were mock-vaccinated with PBS in the two-dose regimen. (**D**) Combination of survival curves shown in (**A**–**C**) to display vaccine efficacy between the different regimens. Statistical significance was determined by Log-rank (Mantel-Cox) test. ns, not significant; *, *p* < 0.05; **, *p* < 0.01; ****, *p* < 0.0001.

CIGM at 10^3^ PFU offered considerable protection without risk to the mice and was therefore used for all downstream experiments. As outlined in Figure 1A, BL6 mice were administered two doses of CIGM; YFV-17D was used as a vaccine control. Administering two doses of CIGM resulted in a 70% survival rate (*n* = 20), compared to the 11% survival rate of a control cohort vaccinated with two doses of 10^3^ PFU YFV-17D (*n* = 9) (Figure 5A). All mice in the control group exhibited signs of disease upon DTV challenge with 8/9 mice succumbing to infection. However, administering a second dose of CIGM did not significantly improve protection from WT DTV challenge relative to a single dose, with a survival rate of 70% and 60%, respectively (Figure 5B). Weight graphs and symptom characterization for the mice administered two-dose vaccinations are presented in Figures S6B,C and S7B,C.

We next assessed the efficacy of a protein boost using the DTV EDIII. In the prime-boost vaccination scheme, BL6 mice were initially vaccinated with CIGM or DTV EDIII, followed by a boost with DTV EDIII. Mice that received the attenuated virus followed by a protein boost were 100% protected from mortality (Figure 5C) and displayed no signs of disease other than transient weight loss in one mouse (Figures S6D and S7D). Of mice that received the DTV EDIII in a homologous prime-boost scheme, 15/17 were protected from morbidity and mortality after challenge (Figure 5C, Figures S6E and S7E). In contrast, all mice of the control cohort that were mock vaccinated with PBS showed signs of disease after DTV challenge, with a survival rate of 20% (Figures S6A and S7A). All experimental cohorts and vaccination regimens are compared in Figure 5D. Despite the small number of animals per cohort (*n* = 15–20), the results suggest that the CIGM YFV-17D-DTV chimeric virus vaccine provides significant protection from lethal DTV disease, with a heterologous prime-boost vaccination using the attenuated virus followed by a EDIII protein boost providing superior protection.

### 3.7. Differences in DTV Levels Post-Challenge after Vaccination

**Figure 6:**
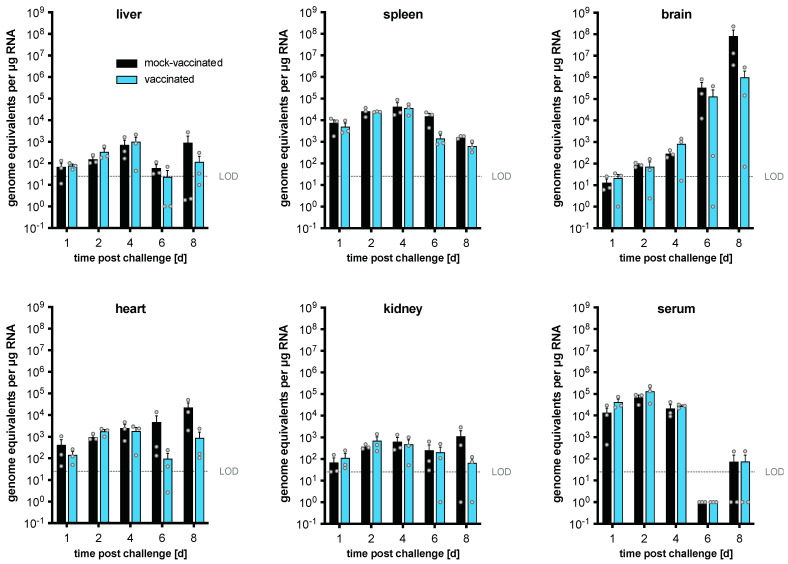
In vivo replication kinetics of WT DTV in naïve and vaccinated BL6 mice. BL6 mice were vaccinated with 10^3^ PFU of CIGM (or PBS as control) in a two-dose regimen, followed by lethal WT DTV challenge 28 d after the last vaccination. At one, two, four, six, and eight days post challenge, groups of three mice were sacrificed to harvest sera and organs, including liver, spleen, brain, heart, and kidney. Viral RNA was extracted and quantified by RT-qPCR. The limit of detection (LOD) is 25 genome equivalents per μg RNA. Graphs are organized by tissue and each grey dot represents the mean viral genome equivalents from an individual mouse from three technical replicates. Bars represent mean values from *n* = 3 mice; error bars indicate the SEM. None of the observed differences between vaccinated or mock-vaccinated mice were statistically significant.

To identify when and where the replication of DTV is restricted in vaccinated versus naïve mice, we resected tissues at different days (one, two, four, six, and eight) post DTV challenge (*n* = 3 per time point). We compared mice that received CIGM in a two-dose regimen to mice that were mock vaccinated with PBS (Figure 6). Viral genome copies consistently increased over time in the brain, which is likely responsible for the observed disease signs such as tremors and paralysis. Viral genome copies in the spleen peaked on day four before slowly decreasing. Virus RNA was also detected in the liver and kidney, though at low levels. Viral RNA was detectable in the serum up to day four.

Differences between viral RNA levels in the unvaccinated and vaccinated cohorts were not statistically significant. However, upon looking at the results from individual vaccinated mice in the later time points (6–8 dpi), a large variation in viral RNA levels in the brain was observed, possibly suggesting that in some mice, vaccination allows for the improved control of virus replication in this highly permissive tissue.

We also determined viral RNA levels in the same tissues shown in Figure 6 on days one, two, four, six, and eight (*n* = 3 per time point) after DTV challenge of mice vaccinated with the heterologous prime-boost regimen of CIGM and DTV EDIII. Interestingly, viral RNA was undetectable at any time point in any of the tissues of the mice except for a low level (6.6 × 10^2^ genome equivalents/μg RNA) in spleen tissue harvested at day two post-infection in one mouse. These data are consistent with the survival seen with this vaccination regimen (Figure 5C), and with the absence of any signs of disease (Figure S7D) or weight loss (Figure S6D), which underscores the strength of this vaccination regimen.

## 4. Discussion

We have successfully established a murine model for POWV disease in adult C57BL/6 mice in our laboratory to assess the efficacy of POWV vaccine candidates. In our initial approach to generate a POWV vaccine candidate, we recoded the virus genome to enrich CpG and UpA dinucleotides, attempting to increase its susceptibility to ZAP or other innate immune responses. All recoded viruses were modestly impaired relative to WT DTV in WT HEK-293T cells. However, replication could only be partially rescued in cells that lacked ZAP (Figure 2 and Figure S2). The attenuation of both CpG- and UpA-high viruses was previously shown to be reversed in ZAP and OAS3/RNase L KO cell lines [35,48,49]. Both CpG- and UpA-high RNA sequences have been shown to bind to ZAP with high affinity in the context of echovirus 7 and influenza A viruses [50]. To our knowledge, no evidence exists that the attenuation of UpA-high mutants is mediated through translational suppression or accelerated RNA degradation. Due to their extensively shared phenotypes, it is possible that unknown mechanisms mediating CpG-high virus restriction may be shared with those restricting UpA-high mutants. UpA-high restriction may also be mediated through other complementary or effector pathways such as KHNYN or TRIM25, previously shown for HIV [51] and SINV [52].

Nevertheless, this observed potential attenuation in vitro did not translate in vivo, and all mice infected with these viruses succumbed to infection (Figure S3). After the design and construction of the recoded DTV mutants, it was reported that the specific spacing of the CpG motifs is important for ZAP-mediated viral restriction [15]. In that work, the authors found that optimal spacing between the CpG dinucleotides to enable ZAP accessibility was between 15 and 32 nucleotides. Increasing the number of A nucleotides in the spacer region was also helpful towards attenuation. They were successful in attenuating a virus by adding 32–48 CpG dinucleotides for a 1 kb segment with an average spacing of 19 nt while increasing the number of A nucleotides from 254 to 386. In our recoded mutants, only 20, 21, 24, and 24 of the CpG motifs for CpG mutant viruses R1-CpG-1, R1-CpG-2, R2-CpG-1, and R2-CpG-2, respectively, were of the reported optimal spacing, with an average spacing of only 13.4, 10.6, 14.4, and 10.9 nt, respectively. We also did not increase the content of A residues between the CpG motifs. Our study only increased CpG or UpA frequencies in specific regions and did not account for spacing and A content, likely explaining the lack of attenuation. Given this new information, additional recoded DTV vaccine candidates could be designed and tested in future experiments.

In our second approach for a POWV vaccine candidate, we replaced the prM and E structural genes of the YFV-17D vaccine strain with those derived from DTV. Interestingly, the chimeric viruses, while only containing the prM and E proteins of DTV, replicated with slower kinetics, similar to DTV, while exhibiting the cytopathic phenotype of YFV by producing sharp plaques (Figure 3A,B, Figure 4A,B and Figure S4). To further reduce the virulence observed in vivo, we mutated a glycosylation motif within the YFV-17D NS1, which has been shown to attenuate YFV [47]. This chimeric virus, named CIGM, protected 70% of mice from a lethal challenge with DTV when given in a two-dose vaccination scheme (Figure 5A). Although higher doses may provide further protection, CIGM was not sufficiently attenuated to allow for this, since one mouse succumbed after vaccination with the 10^4^ PFU dose of CIGM. After sequencing the viral genome, we found the mutation disrupting the glycosylation site in NS1 had not reverted back to the WT sequence. In a similar circumstance, a mouse succumbed to the YFV-17D vaccine, suggesting that the vaccine strain itself may cause disease in rare cases. It is conceivable that CIGM could be further attenuated by removing a second glycosylation site within NS1, which may reduce its virulence, allowing the assessment of higher vaccine doses. Substituting additional proteins from DTV into the YFV-17D backbone could also potentially lead to improved protection.

Interestingly, the heterologous prime-boost vaccination regimen pairing the live-attenuated CIGM vaccine with a protein boost, consisting of the domain III of the E protein, increased the survival rate to 100%, with no signs of disease (Figure 5C, Figures S6D and S7D), and with essentially no DTV RNA detected in any tissue examined after challenge. Two doses of the DTV EDIII protein were also protective, conferring an 88% survival rate upon challenge with DTV (Figure 5C). It is uncertain how the length of protection and the breadth of antibody generation and/or T-cell responses might vary between the live-attenuated chimeric virus vaccine versus the protein subunit vaccine. Of note, we were unable to detect the CIGM vaccine in any of the mice post-vaccination, suggesting that replication may be very limited and that a protein boost may compensate for this. Altogether, the mechanism(s) by which CIGM elicits a protective immune response is not understood and should be evaluated in future studies.

Finally, it will be of interest to test the breadth of protection elicited by CIGM and its ability to protect mice from lethal infection with POWV (lineage 1). POWV and DTV share 94% amino acid sequence identity [6], so it is hypothesized that a live-attenuated vaccine developed for one lineage could protect against the other.

## Figures and Tables

**Table 1:** CpG and UpA dinucleotide composition in recoded regions of DTV.

Region	Sequence Composition	G + C Content	Total CpG (Change)	Total UpA (Change)	Ratio CpG	Ratio UpA
Full length	Native	0.5274	380	281	0.51	0.46
R1 *	WT	0.5333	56 (-)	49 (-)	0.5231	0.5848
	CDLR	0.5333	56 (0)	49 (0)	0.5231	0.5848
	CpG-1	0.533	106 (+50)	49 (0)	0.9901	0.5848
	CpG-2	0.5333	133 (+77)	49 (0)	1.2423	0.5848
	UpA-1	0.5333	56 (0)	83 (+34)	0.5231	0.9907
	UpA-2	0.5333	56 (0)	115 (+66)	0.5231	1.3726
R2 *	WT	0.5213	54	33	0.5918	0.4238
	CDLR	0.5213	54	33	0.5918	0.4238
	CpG-1	0.5213	90 (+36)	33 (0)	0.9863	0.4238
	CpG-2	0.5213	121 (+67)	33 (0)	1.326	0.4238
	UpA-1	0.5213	54 (0)	77 (+44)	0.5918	0.989
	UpA-2	0.5213	54 (0)	100 (+67)	0.5918	1.2844

* Fragment sizes: R1: 1547 bp and R2: 1385 bp.

## Data Availability

Data is contained within the article or supplementary material.

## Supplementary Materials

The following supporting information can be downloaded at: https://www.mdpi.com/article/10.3390/vaccines11030612/s1, Supplementary File S1; Figure S1. Schematics of the vaccine candidates; Figure S2 (related to Figure 2). Replication kinetics of CpG and UpA recoded DTV constructs after low MOI infection; Figure S3 (related to Figure 2). Infection of BL6 mice with CpG and UpA recoded DTV viruses; Figure S4. Plaque phenotypes of parental and YFV-17D-DTV Chimera I viruses; Figure S5 (related to Figure 4). Disease scores of BL6 mice vaccinated with a single dose of CIGM followed by lethal DTV challenge; Figure S6 (related to Figure 5). Normalized weight of BL6 mice vaccinated with the two-dose regimens followed by lethal DTV challenge; Figure S7 (related to Figure 5). Disease scores of BL6 mice vaccinated with two-dose regimens followed by lethal DTV challenge.
