# Supplementary material for: Characterization of Live-Attenuated Powassan Virus Vaccine Candidates Identifies an Efficacious Prime-Boost Strategy for Mitigating Powassan Virus Disease in a Murine Model
Source: Vaccines (Basel). 2023 Mar 8;11(3):612. doi: 10.3390/vaccines11030612 (PMC10058527; doi:10.3390/vaccines11030612)
Supplement: Supplementary file 1 [file vaccines-11-00612-s001.zip › Supplemental Figures_revised_FINAL2.pdf]

Supplemental Figure 1

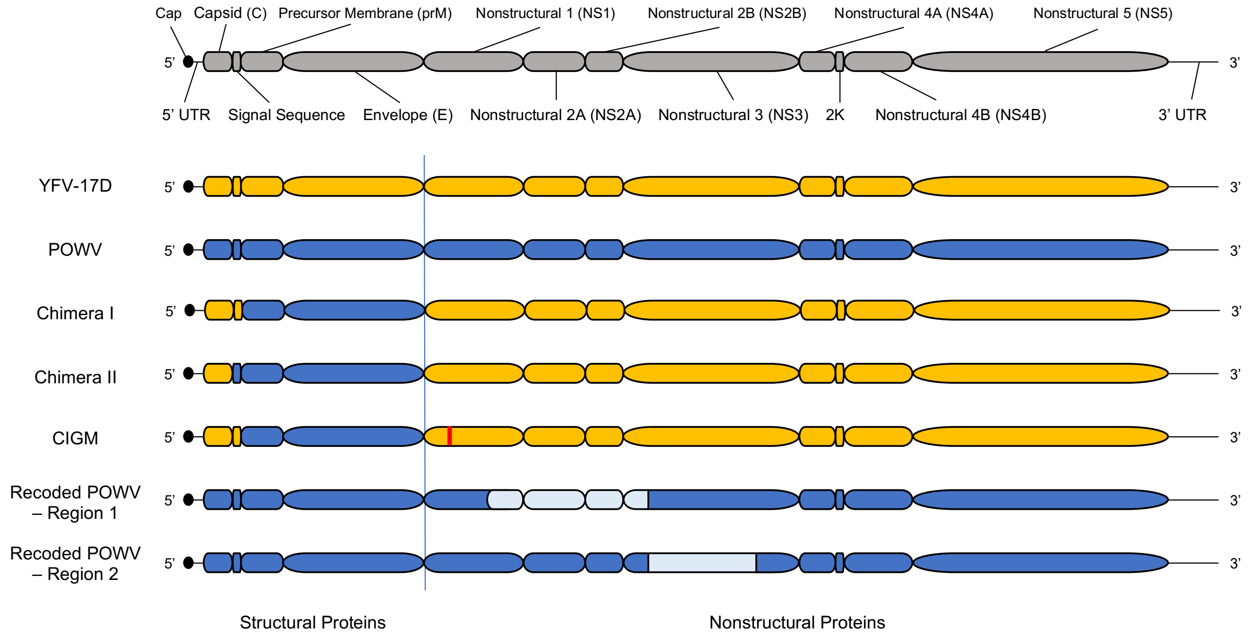

Supplemental Figure S1. Schematics of the vaccine candidates.
A schematic of a generic flavivirus genome is shown at the top (gray) with the relevant features labelled. Below are schematics of YFV-17D (yellow), POWV (blue), Chimera I, Chimera II, CIGM, and the Region 1 and Region 2 recoded POWVs. The demarcation between the structural and nonstructural proteins is indicated by a vertical blue line; red indicates the location of the YFV NS1 glycosylation site mutation; light blue indicates the POWV regions recoded to increase CpG or UpA dinucleotide content.

Supplemental Figure 2

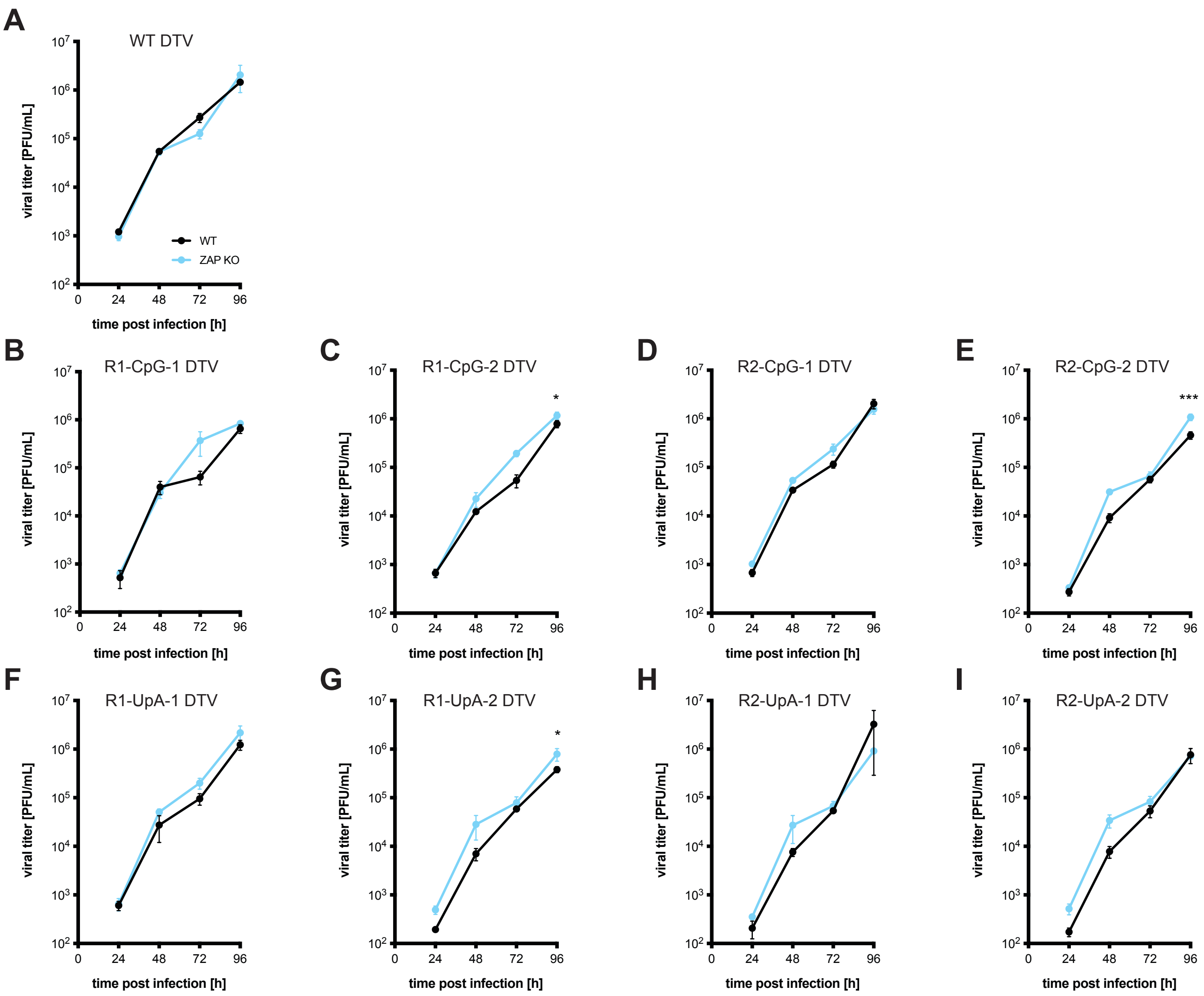

Supplemental Figure S2 (related to Figure 2). **Replication kinetics of CpG and UpA recoded DTV constructs after low MOI infection.**
WT and ZAP KO HEK-293T cells were infected (MOI = 0.01 PFU/cell) with (A) WT DTV, (B - E) CpG recoded viruses, and (F - I) UpA recoded viruses. Supernatants were harvested daily for 4 d and infectious virus was titered by plaque assay. Mean titers of triplicate samples are plotted; error bars indicate the SD. *, $p<0.05$; ***, $p<0.001$, 2-way ANOVA with Šidák's multiple comparisons test.

Supplemental Figure 3

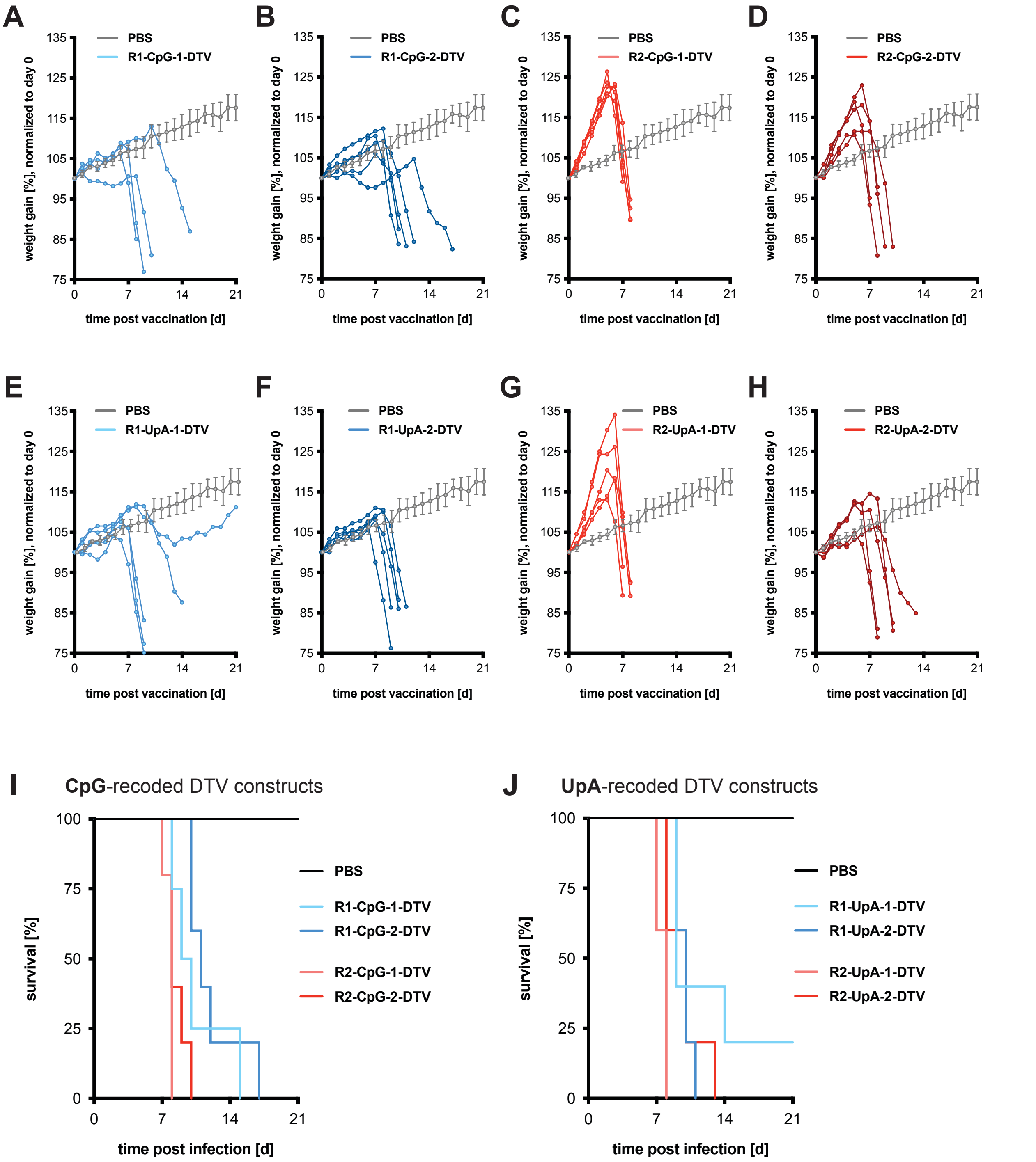

Supplemental Figure S3 (related to Figure 2)**. Infection of BL6 mice with CpG and UpA recoded DTV viruses.** Four-week-old BL6 mice (n = 5 per cohort) were infected with 10^3 PFU of CpG recoded viruses (A - D) or UpA recoded viruses (E - H). The average weight gain of mock-infected control mice (n = 5) is shown in grey in each graph. The survival of mice infected in (A - D) with CpG recoded viruses (I) or of mice infected in (E - H) with UpA recoded viruses (J) are plotted.

Supplemental Figure 4

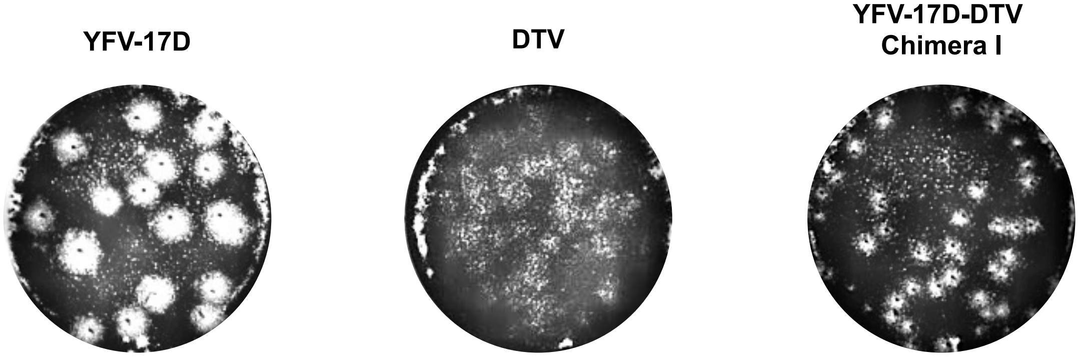

Supplemental Figure S4. Plaque phenotypes of parental and YFV-17D-DTV Chimera I viruses.
Example plaques for YFV-17D, DTV and YFV-17D-DTV Chimera I in Huh-7.5 cells are shown.

Supplemental Figure 5

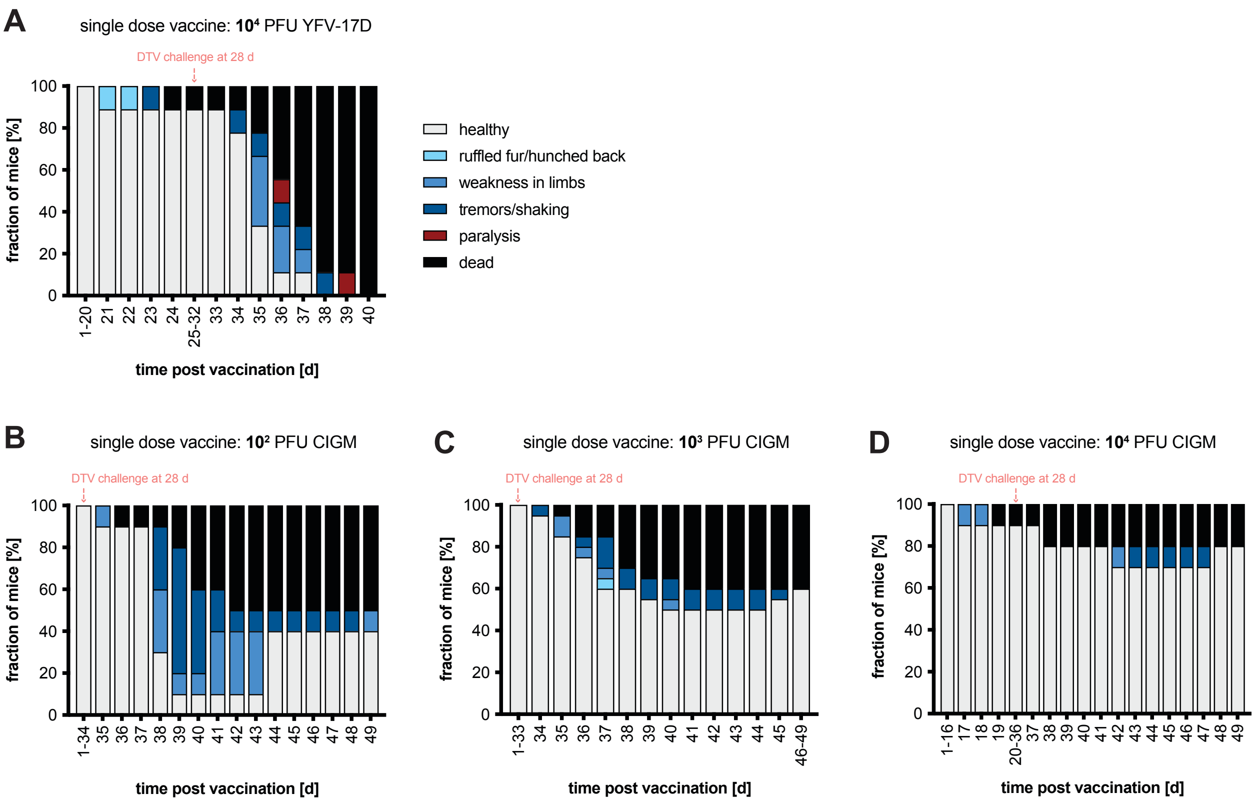

Supplemental Figure S5 (related to Figure 4)**. Disease scores of BL6 mice vaccinated with a single dose of CIGM followed by lethal DTV challenge**.

BL6 mice were vaccinated with CIGM or YFV-17D as indicated and were challenged with DTV as described in Figure 4C. The animals were assessed daily to characterize disease signs ordered by increasing severity of POWV disease as indicated and detailed in **Materials and Methods**. Of note, one mouse in both cohorts infected with 10^4 PFU YFV-17D control and CIGM died after vaccination prior to lethal DTV challenge.

Supplemental Figure 6

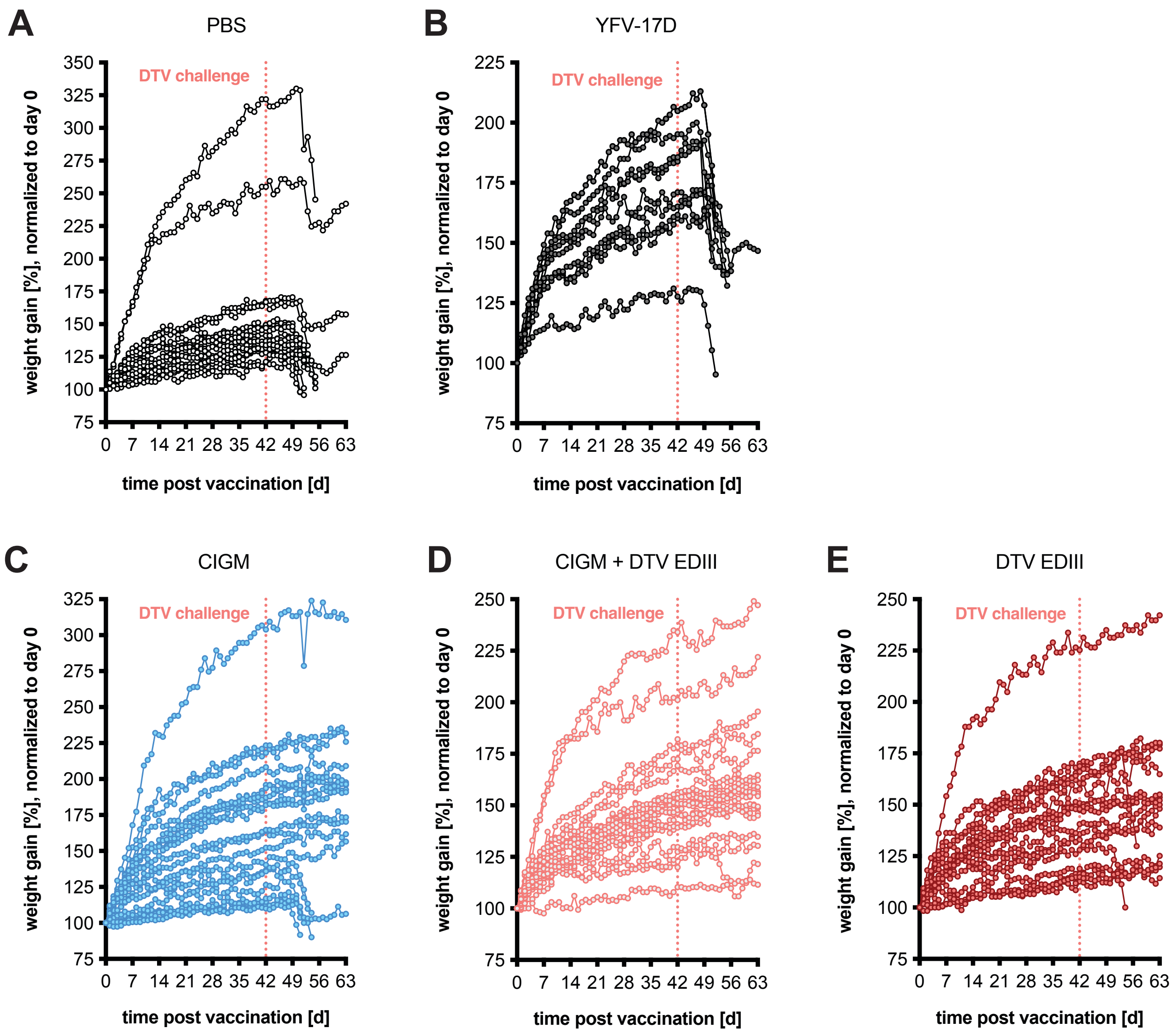

Supplemental Figure S6 (related to Figure 5)**. Normalized weight of BL6 mice vaccinated with the two-dose regimens followed by lethal DTV challenge.**

BL6 mice were vaccinated and challenged with DTV as described in **Figure 5A** and **5C**. Briefly, mice were vaccinated on day 0, boosted on day 14, and challenged with WT DTV on day 42 as indicated. The animals were monitored daily for weight loss and disease signs and sacrificed upon reaching humane endpoints. Weights were normalized to day 0 prior to the initial vaccination. Results after vaccination with: (A) two doses PBS (n = 15), (B) two doses 10^3 PFU YFV-17D (n = 9), (C) two doses 10^3 PFU CIGM (n = 20), (D) 10^3 PFU CIGM followed by 25 ng of DTV EDIII (n = 19), and (E) two doses 25 ng DTV EDIII (n = 17) are plotted.

Supplemental Figure 7

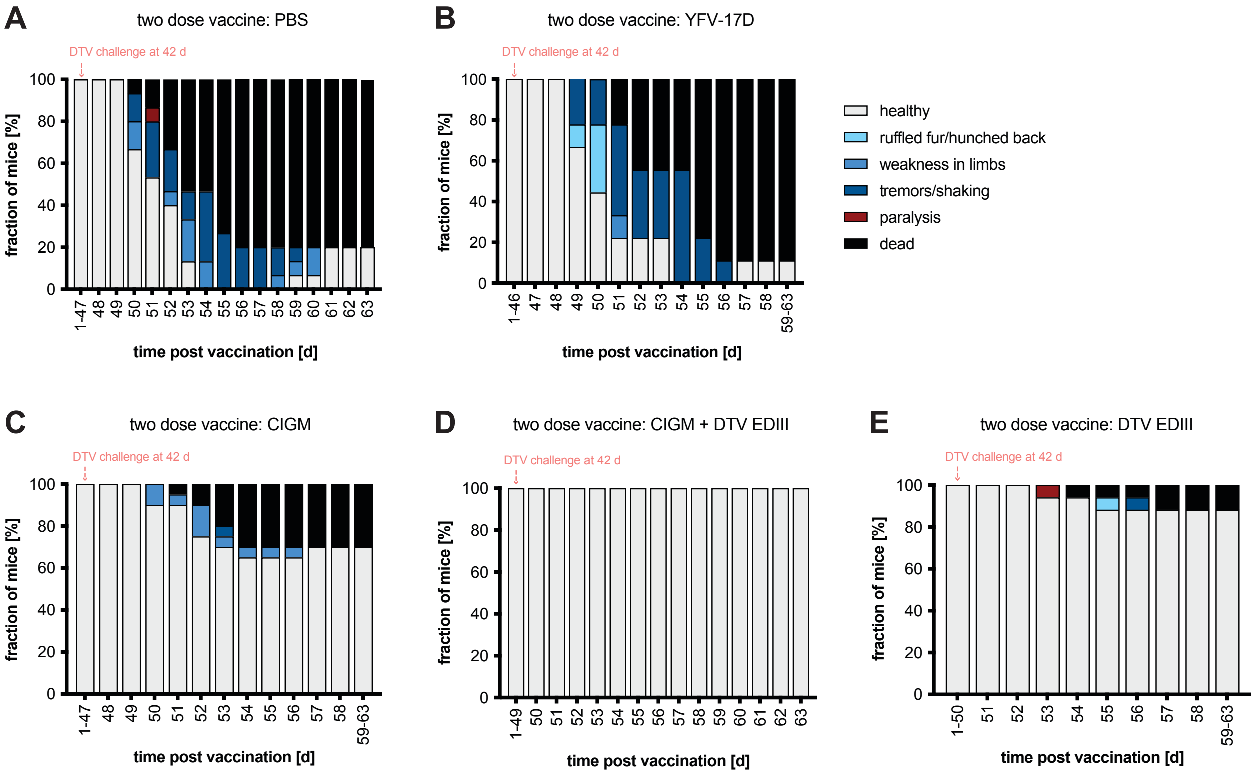

Supplemental Figure S7 (related to Figure 5). **Disease scores of BL6 mice vaccinated with two-dose regimens followed by lethal DTV challenge.**

BL6 mice were vaccinated and challenged with DTV as indicated and described in **Figure 5A** and **5C**. The animals were assessed daily to characterize disease signs ordered by increasing severity of POWV disease as indicated and detailed in **Materials and Methods**. Results after vaccination with: (A) two doses PBS (n = 15), (B) two doses 10^3 PFU YFV-17D (n = 9), (C) two doses 10^3 PFU CIGM (n = 20), (D) 10^3 PFU CIGM followed by 25 ng of DTV EDIII (n = 19), and (E) two doses 25 ng DTV EDIII (n = 17) are plotted.
