# Supplementary material for: Characterization of Live-Attenuated Powassan Virus Vaccine Candidates Identifies an Efficacious Prime-Boost Strategy for Mitigating Powassan Virus Disease in a Murine Model
Source: Vaccines (Basel). 2023 Mar 8;11(3):612. doi: 10.3390/vaccines11030612 (PMC10058527; doi:10.3390/vaccines11030612)
Supplement: Supplementary file 1 [file vaccines-11-00612-s001.zip › Supplementary File 1.pdf]

Gene Fragment Sequences

GB2a
>Geneblock 2a (prME) complete sequence 1630 bp
GTCGACGCGGCCGCTAGCGATGACCCTGCTGATTGGTTCGCTGACCATTTCCGGGTGCG
GGACGGCGTTACCAGAAACTCAGAAGGTTCGTCCAACCAAACCGACTCTGACGGCAGTT
TACGAGAGAGATGATAGGGTCTGCTTCAGTAAGCCAGATGCTACACAATTAGGCTTGTAC
ATATTGTCGTTAGAACGCGGCTACAATTAATACATAACCTTATGTATCATACACATACGATT
TAGGTGACACTATAGAGTAAATCCTGTGTGCTAATTGAGGTGCATTGGTCTGCAAATCGA
GTTGCTAGGCAATAAACACATTTGGATTAATTTTAATCGTTCGTTGAGCGATTAGCAGAGA
ACTGACCAGAACATGTCTGGTCGTAAAGCTCAGGGAAAAACCCTGGGCGTCAATATGGTA
CGACGAGGAGTTCGCTCCTTGTCAAACAAAATAAAACAAAAAACAAAACAAATTGGAAACA
GACCTGGACCTTCAAGAGGTGTTCAAGGATTTATCTTTTTCTTTTTGTTCAACATTTTGACT
GGAAAAAAGATCACAGCCCACCTAAAGAGGTTGTGGAAAATGCTGGACCCAAGACAAGG
CTTGGCTGTTCTAAGGAAAGTCAAGAGAGTGGTGGCCAGTTTGATGAGAGGATTGTCCTC
AAGGAAACGCCGTTCCCATGATGTTCTGACTGTGCAATTCCTAATTTTGGGAATGCTGTTG
ATGACGGGTGGAACTTCCATCCATCGGGATAGAGAGGGATACATGGTCATGCGAGCTAG
CGGAAGAGATGCAGCAAGTCAAGTTAGAGTTCAAAACGGAACATGCGTTATTTTGGCAAC
GGACATGGGAGAATGGTGTGAAGATTCAATAACCTACTCCTGTGTTACGATTGACCAGGA
AGAAGAACCTGTTGATGTGGACTGTTTTTGCCGAGGTGTTGACAGAGTCAAGTTGGAGTA
CGGACGCTGTGGTAGACAGGCTGGATTCAGGGGGAGAAGGTCTGTGGTCATCCCGACA
CATGCACAAAAGGACATGGTTGGGCGAGGACACGCATGGCTCAAGGGTGACAACATTCG
AGATCATGTTACCCGTGTCGAGGGCTGGATGTGGAAAAACAAGCTCTTGACAGTTGCCGT
AGTGGCTTTGGCTTGGCTCATGCTGGATAGTTGGATGGCTAGGGTGACTGTGATCCTTCT
GGCGCTGAGCCTTGGGCCCGTATATGCCACGAGATGCACGCACCTTGAGAACAGGGACT
TTGTAACAGGAGTACAGGGAACCACTAGGGTGTCCCTGGTTCTGGAGCTTGGGGGATGT
GTGACCATCACTGCTGAGGGCAAGCCGTCCATTGACGTATGGCTGGAGGACATTTTCCAA
GAAAGTCCCGCTGAAACCAGAGAATACTGTCTACACGCCAAGCTTTCCAATACAAAAGTA
GAGGCTCGCTGCCCTACCACTGGGCCAGCTACGCTTCCAGAGGAGCACCAGGCCAACAT
GGTGTGTAAGAGAGACCAGAGCGACCGCGGATGGGGAAACCACTGTGGATTTTTCGGGA
AAGGAAGCATCGTGGCCTGTGCCAAGTTTGAATGTGAGGAAGCAAAAAAGGCTGTAGGT
CATGTTTATGACTCA

GB2b
>Geneblock 2b (SigprME) complete sequence 1630 bp
GTCGACGCGGCCGCTAGCGATGACCCTGCTGATTGGTTCGCTGACCATTTCCGGGTGCG
GGACGGCGTTACCAGAAACTCAGAAGGTTCGTCCAACCAAACCGACTCTGACGGCAGTT
TACGAGAGAGATGATAGGGTCTGCTTCAGTAAGCCAGATGCTACACAATTAGGCTTGTAC
ATATTGTCGTTAGAACGCGGCTACAATTAATACATAACCTTATGTATCATACACATACGATT
TAGGTGACACTATAGAGTAAATCCTGTGTGCTAATTGAGGTGCATTGGTCTGCAAATCGA
GTTGCTAGGCAATAAACACATTTGGATTAATTTTAATCGTTCGTTGAGCGATTAGCAGAGA
ACTGACCAGAACATGTCTGGTCGTAAAGCTCAGGGAAAAACCCTGGGCGTCAATATGGTA
CGACGAGGAGTTCGCTCCTTGTCAAACAAAATAAAACAAAAAACAAAACAAATTGGAAACA
GACCTGGACCTTCAAGAGGTGTTCAAGGATTTATCTTTTTCTTTTTGTTCAACATTTTGACT
GGAAAAAAGATCACAGCCCACCTAAAGAGGTTGTGGAAAATGCTGGACCCAAGACAAGG
CTTGGCTGTTCTAAGGAAAGTCAAGAGAGTGGTGGCCAGTTTGATGAGAGGATTGTCCTC

AAGGAAACGCCGTTCAGGCGTTGACTGGACATGGACCTTCTTAGTTATGGCTTTGATGAC
TATGGCCATGGCAACTTCCATCCATCGGGATAGAGAGGGATACATGGTCATGCGAGCTA
GCGGAAGAGATGCAGCAAGTCAAGTTAGAGTTCAAAACGGAACATGCGTTATTTTGGCAA
CGGACATGGGAGAATGGTGTGAAGATTCAATAACCTACTCCTGTGTTACGATTGACCAGG
AAGAAGAACCTGTTGATGTGGACTGTTTTTGCCGAGGTGTTGACAGAGTCAAGTTGGAGT
ACGGACGCTGTGGTAGACAGGCTGGATTCAGGGGGAGAAGGTCTGTGGTCATCCCGACA
CATGCACAAAAGGACATGGTTGGGCGAGGACACGCATGGCTCAAGGGTGACAACATTCG
AGATCATGTTACCCGTGTCGAGGGCTGGATGTGGAAAAACAAGCTCTTGACAGTTGCCGT
AGTGGCTTTGGCTTGGCTCATGCTGGATAGTTGGATGGCTAGGGTGACTGTGATCCTTCT
GGCGCTGAGCCTTGGGCCCGTATATGCCACGAGATGCACGCACCTTGAGAACAGGGACT
TTGTAACAGGAGTACAGGGAACCACTAGGGTGTCCCTGGTTCTGGAGCTTGGGGGATGT
GTGACCATCACTGCTGAGGGCAAGCCGTCCATTGACGTATGGCTGGAGGACATTTTCCAA
GAAAGTCCCGCTGAAACCAGAGAATACTGTCTACACGCCAAGCTTTCCAATACAAAAGTA
GAGGCTCGCTGCCCTACCACTGGGCCAGCTACGCTTCCAGAGGAGCACCAGGCCAACAT
GGTGTGTAAGAGAGACCAGAGCGACCGCGGATGGGGAAACCACTGTGGATTTTTCGGGA
AAGGAAGCATCGTGGCCTGTGCCAAGTTTGAATGTGAGGAAGCAAAAAAGGCTGTAGGT
CATGTTTATGACTCA

GB3
>Geneblock 3 (DTV E into YF NS1) complete sequence 1656 bp
GCCTGTGCCAAGTTTGAATGTGAGGAAGCAAAAAAGGCTGTAGGTCATGTTTATGACTCA
ACAAAAATCACATATGTTGTCAAGGTCGAGCCCCATACGGGGGATTACCAAGCTGCAAAT
GAGACCAATGAGAACAGGAAAACGGCACAGTTCACAGTGGCATCCGAGAAGGTGATCTT
GGACCTAGGAGACTATGGAGATGTGTCATTGACATGCAAGGTGGCAAGTGGAATTGATGT
CGCCCAGACTGTGGTGATGTCGCTTGGCAGTAGCAAAGATCATCTGCCTTCGGCATGGC
AGTTGCATCGTGACTGGTTCGAGGACCTAGCCCTGCCTTGGAAACATAAAGATAATCAAG
ATTGGAACAGTGTGGAGAAACTTGTGGAGTTCGGGCCACCTCATGCTGTAAAAATGGACA
TCTTTAATCTGGGGGATCAGACTGCTGTTCTGCTCAAATCACTGGCTGGAGTCCCACTGG
CAAGTGTGGACAACCAGAAATATCACTTGAAAAGTGGTCATGTCACCTGTGATGTGGGGC
TTGAAAAGTTGAAGCTGAAAGGCACAACTTACTCCATGTGTGACAAAACAAAGTTCAAATG
GAAAAGAGTTCCTGTGGACAGTGGTCATGATACAGTGGTCATGGAGGTGTCTTACACAGG
GAGTGACAAGCCCTGCCGAATTCCAGTGCGAGCAGTGGCGCACGGTGTCCCGACAATCA
ATGTGGCCATGCTCATTACCCCCAACCCAACCATTGAAACAAGTGGAGGAGGATTCATAG
AGATGCAGTTGCCACCTGGGGACAACATTATCTATGTGGGGGATCTTAGTCAGCAGTGGT
TCCAGAAAGGCAGCACCATAGGTAGAATGTTCGAAAAGACTCGCAAGGGTTTGGAAAGAC
TCTCAGTGGTTGGAGAACACGCATGGGACTTTGGTTCAGTGGGTGGAATCCTGTCGTCAG
TTGGAAAGGCCATCCATACGGTGTTGGGGGGGGCATTCAACACCCTTTTTGGGGGAGTT
GGGTTCATCCCTAAGATGCTGTTGGGAGTCGCCTTGGTGTGGTTGGGGCTTAACGCTAG
GAATCCAACAATGTCTATGACTTTTCTTGTCGTGGGGGCCCTGACCTTGATGATGACAATG
GGAGTCGGAGCAGATCAAGGATGCGCCATCAACTTTGGCAAGAGAGAGCTCAAGTGCGG
AGATGGTATCTTCATATTTAGAGACTCTGATGACTGGCTGAACAAGTACTCATACTATCCA
GAAGATCCTGTGAAGCTTGCATCAATAGTGAAAGCCTCTTTTGAAGAAGGGAAGTGTGGC
CTAAATTCAGTTGACTCCCTTGAGCATGAGATGTGGAGAAGCAGGGCAGATGAGATCAAT
GCCATTTTTGAGGAAAACGAGGTGGACATTTCTGTTGTCGTGCAGGATCCAAAGAATGTT
TACCAGAGAGGAACTCATCCATTTTCCAGAATTCGGGATGGTCTGCAGTATGGTTGGAAG
ACTTGGGGTAAGAACCTTGTGTTCTCCCCAGGGAGGAAGAATGGAAGCTTCATCATAGAT

GGAAAGTCCAGGAAAGAATGCCCGTTTTCAAACCGGGTCTGGAATTCTTTCCAGATAGAG
GAGTTTGGGACGGGAGTGTTCACCACACGCGTGTACATG

Gene Fragments obtained in plasmids for Powassan virus recoding

>R1_CDLR
CTAAATTGTAAGCGTTAATATTTTGTTAAAATTCGCGTTAAATTTTTGTTAAATCAGCTCATT
TTTTAACCAATAGGCCGAAATCGGCAAAATCCCTTATAAATCAAAAGAATAGACCGAGATA
GGGTTGAGTGGCCGCTACAGGGCGCTCCCATTCGCCATTCAGGCTGCGCAACTGTTGGG
AAGGGCGTTTCGGTGCGGGCCTCTTCGCTATTACGCCAGCTGGCGAAAGGGGGATGTGC
TGCAAGGCGATTAAGTTGGGTAACGCCAGGGTTTTCCCAGTCACGACGTTGTAAAACGAC
GGCCAGTGAGCGCGACGTAATACGACTCACTATAGGGCGAATTGGCGGAAGGCCGTCAA
GGCCACGTGTCTTGTCCGCTTAATTAACATCGATAATGATGGAGTGTTGGATTCACGCCT
CTTCCTACCCGTCACTCTAGCAGGGCCAAGGTCAAAATACAACAGAATTCCGGGATATAG
TGAGCAGACGAAGGGTCCGTGGGACCAGACTCCACTGAGAGTTGTCCGGGATCATTGTC
CTGGAACAAGCGTGAAGATTGACAGTCAGTGCGACAAGAGGGGAGCTTCAGTCAGAAGC
ACCACAGAGAGTGGAAAAATCATCCCAGAGTGGTGTTGCCGGGCCTGTGAACTGCCACC
GGTGACTTTCCGCAGCGGGACTGACTGTTGGTATGCGATGGAAATCAGACCTGTTCACTC
GCAGGGAGGCCTGGTCAGATCCATGGTAATGGCGGACAACGGGGCTATGCTTAGCGAG
GGTGGAGTACCTGGACTGGTGGCTGTGTTTGTACTGATGGAATTTTTGTTGAGACGGCGA
CCTGGATCTGTCACCTCGATTTTGTGGGGTGGTATACTCATGCTGGGATTGCTTGTTACT
GGACTGGTGGGAGTGGAGGAGATTGTACGCTACTTCATAGCTGTGGGGATCACCTTCCA
CCTGGAGTTGGGGCCAGAGATCATGGCACTTGTGATGCTCCAAGCTGTTTTTAGCATGCG
GACGTGCTATCTCACTGGTTTCCTCACAAAGAAGGTGATTACCACACGTGAAGTGGTGAC
GGTCTATTTCCTGCTGTTGGTCCTGGAGATGGGCATTCCAGAAATGAACCTGGGACATTT
ATGGGAGTGGGCTGATTCCTTGGCAATGGGACTGCTCATAATCAAGGCCAGTTCTATGGA
AGACCGCAAGGGTTTAGGATTCCTATTGGCTGGACTCATGACCCAGCGCCACCTGACTG
CTGTTCATCACGGCTTGATAGTCTTCCTCACTGTGGCATTGGCCGTTGTGGGGCGAAACA
TTTACAATGGGCAAAAAGAGAGGAAAGGATTAAGCTTCACCATCCCTTTGGCCTCCCTAC
TTGGCGGCCCTGGTTCGGGCTTACGCATGTTGGCGTTGTGGGAGTGTCTGAGGGGGCAC
GGCAGACGAAGTCTAAGTGAGCCGCTCACTGTGGTTGGAGTCATGTTGGCTATGGCAAG
TGGACTACTGCGGCACTCTTCCCAGGAGGCTTTGCTGGTCCTATCCGCAGGATCCTTCCT
CATACTGATGTTGATATTGGGCACCCGGAGACTTCAGTTGACGGCGGAGTGGGCGGGCT
TGGCAGAATGGAATCCGGAATTGGTCAACGAAGGGGGAGAGGTTAGCCTAAAGGTGAGG
CAGGATGCCATGGGGAACTTGCACTTGACTGAAGTGGAACGCGAGGAACGCAGGCTGG
CTCTCTGGCTAGTATTCGGCTTGCTGGCGTCGGCTTTCCACTGGTCTGGCATCTTGGTGA
CTATGGGAGCATGGACCCTCTATGAGCTCCTCTCTTCAACCCGCAGAACTGACCTTGTTT
TCTCTGGACAACTCTCAGACCATGGGGAAAGCAGGCCATTTGACATCAAAGACGGGGTAT
ACCGAATATATGCTCCGGGACTCTTATGGGGTCACCGGCGCGCCGGAGCACAAGACTGG
CCTCATGGGCCTTCCGCTCACTGCCCGCTTTCCAGTCGGGAAACCTGTCGTGCCAGCTG
CATTAACATGGTCATAGCTGTTTCCTTGCGTATTGGGCGCTCTCCGCTTCCTCGCTCACTG
ACTCGCTGCGCTCGGTCGTTCGGGTAAAGCCTGGGGTGCCTAATGAGCAAAAGGCCAGC
AAAAGGCCAGGAACCGTAAAAAGGCCGCGTTGCTGGCGTTTTTCCATAGGCTCCGCCCC
CCTGACGAGCATCACAAAAATCGACGCTCAAGTCAGAGGTGGCGAAACCCGACAGGACT
ATAAAGATACCAGGCGTTTCCCCCTGGAAGCTCCCTCGTGCGCTCTCCTGTTCCGACCCT
GCCGCTTACCGGATACCTGTCCGCCTTTCTCCCTTCGGGAAGCGTGGCGCTTTCTCATAG

CTCACGCTGTAGGTATCTCAGTTCGGTGTAGGTCGTTCGCTCCAAGCTGGGCTGTGTGCA
CGAACCCCCCGTTCAGCCCGACCGCTGCGCCTTATCCGGTAACTATCGTCTTGAGTCCAA
CCCGGTAAGACACGACTTATCGCCACTGGCAGCAGCCACTGGTAACAGGATTAGCAGAG
CGAGGTATGTAGGCGGTGCTACAGAGTTCTTGAAGTGGTGGCCTAACTACGGCTACACTA
GAAGAACAGTATTTGGTATCTGCGCTCTGCTGAAGCCAGTTACCTTCGGAAAAAGAGTTG
GTAGCTCTTGATCCGGCAAACAAACCACCGCTGGTAGCGGTGGTTTTTTTGTTTGCAAGC
AGCAGATTACGCGCAGAAAAAAAGGATCTCAAGAAGATCCTTTGATCTTTTCTACGGGGT
CTGACGCTCAGTGGAACGAAAACTCACGTTAAGGGATTTTGGTCATGAGATTATCAAAAA
GGATCTTCACCTAGATCCTTTTAAATTAAAAATGAAGTTTTAAATCAATCTAAAGTATATATG
AGTAAACTTGGTCTGACAGTTACCAATGCTTAATCAGTGAGGCACCTATCTCAGCGATCTG
TCTATTTCGTTCATCCATAGTTGCCTGACTCCCCGTCGTGTAGATAACTACGATACGGGAG
GGCTTACCATCTGGCCCCAGTGCTGCAATGATACCGCGAGAACCACGCTCACCGGCTCC
AGATTTATCAGCAATAAACCAGCCAGCCGGAAGGGCCGAGCGCAGAAGTGGTCCTGCAA
CTTTATCCGCCTCCATCCAGTCTATTAATTGTTGCCGGGAAGCTAGAGTAAGTAGTTCGCC
AGTTAATAGTTTGCGCAACGTTGTTGCCATTGCTACAGGCATCGTGGTGTCACGCTCGTC
GTTTGGTATGGCTTCATTCAGCTCCGGTTCCCAACGATCAAGGCGAGTTACATGATCCCC
CATGTTGTGCAAAAAAGCGGTTAGCTCCTTCGGTCCTCCGATCGTTGTCAGAAGTAAGTT
GGCCGCAGTGTTATCACTCATGGTTATGGCAGCACTGCATAATTCTCTTACTGTCATGCCA
TCCGTAAGATGCTTTTCTGTGACTGGTGAGTACTCAACCAAGTCATTCTGAGAATAGTGTA
TGCGGCGACCGAGTTGCTCTTGCCCGGCGTCAATACGGGATAATACCGCGCCACATAGC
AGAACTTTAAAAGTGCTCATCATTGGAAAACGTTCTTCGGGGCGAAAACTCTCAAGGATCT
TACCGCTGTTGAGATCCAGTTCGATGTAACCCACTCGTGCACCCAACTGATCTTCAGCAT
CTTTTACTTTCACCAGCGTTTCTGGGTGAGCAAAAACAGGAAGGCAAAATGCCGCAAAAA
AGGGAATAAGGGCGACACGGAAATGTTGAATACTCATACTCTTCCTTTTTCAATATTATTG
AAGCATTTATCAGGGTTATTGTCTCATGAGCGGATACATATTTGAATGTATTTAGAAAAATA
AACAAATAGGGGTTCCGCGCACATTTCCCCGAAAAGTGCCAC

>R2_CDLR
CTAAATTGTAAGCGTTAATATTTTGTTAAAATTCGCGTTAAATTTTTGTTAAATCAGCTCATT
TTTTAACCAATAGGCCGAAATCGGCAAAATCCCTTATAAATCAAAAGAATAGACCGAGATA
GGGTTGAGTGGCCGCTACAGGGCGCTCCCATTCGCCATTCAGGCTGCGCAACTGTTGGG
AAGGGCGTTTCGGTGCGGGCCTCTTCGCTATTACGCCAGCTGGCGAAAGGGGGATGTGC
TGCAAGGCGATTAAGTTGGGTAACGCCAGGGTTTTCCCAGTCACGACGTTGTAAAACGAC
GGCCAGTGAGCGCGACGTAATACGACTCACTATAGGGCGAATTGGCGGAAGGCCGTCAA
GGCCACGTGTCTTGTCCGCGGTACCGGTCACCGGCAGATCGGAGTGGGATATGGCACC
AAAGGTGTCCTCCACACTATGTGGCATGTGACCAGAGGAGCTGCACTCAGTGTTGAAGG
AGCCGTTTCCGGACCTTATTGGGCTGATGTGCGTGAGGATGTGGTGTGCTACGGAGGGG
CATGGAGTCTGGAAGGCAAATGGGGAGGGGAGGTTGTGCAGGTCCACGCCTTTCCTCCA
GATAGTGGCCACAAAGTTCATCAATGTCAACCCGGCAAACTGAATCTTGAAGGAGGGCGT
GTAATGGGTGCAATCCCAATAGATCTACCCCGTGGCACATCGGGGAGCCCCATCATCAAT
GCTCAGGGGATTGTCCTGGGTCTTTACGGGAACGGACTGAAATCAAACGATGTCTACATC
AGCAGCATTGCGCAAGGAAGCGTTGAAAAGAGCCGACCAGATATGCCCTTGGCAGTTCA
GGGGGGCAGATGGACATCCAAGGGTAGCATCACCGTTTTGGATATGCATCCTGGTTCAG
GAAAGACACACAGAGTTCTCCCAGAGCTGATCCGAGAATGCATTGATAAAAGGCTGAGAA
CTGTTGTGCTGGCTCCCACACGTGTTGTCTTAAAGGAAATGGAAAGAGCACTTCAGGGCA
AGCGTGTCAAATTCCATTCAGCGGCAGTTGACAACACATCATCTTCATCTGGCGCCATCG

TGGATGTCATGTGCCACGCCACATATGTGAATCGCCGACTGTTGCCCCAAGGGAGACAG
AATTGGGAAGTGGCCATCATGGATGAGGCACACTGGACTGACCCCCACTCCATAGCTGC
ACGGGGTCATTTGTATTCCTTGGCCAAAGAAAATAGATGCGCTTTTGTTTTGATGACGGCC
ACCCCGCCCGGAAAAAGTGAGGCTTTTCCAGAATCAAAAGGAGCAATAGTCAGTGAAGAA
CGCCCCATACCTGATGGGGAGTGGAGAGATGGGTTTGATTGGGTGACAGAATTTGAGGG
CCGAACCGCATGGTTCGTATCGTCAATAGCAAAAGGAGGGGCGATTGCTCGCACGTTGA
GGCAGAAAGGGAAAAGTGTGATCTGCTTGAACAGCAAGACTTTTGACAAGGACTATGGCA
GAGTGCGTGAAGAAAAACCGGACTTTGTTGTCACTACGGACATCAGCGAGATGGGGGCT
AACCTGGACGTTCACAGAGTAATAGATGGTAGGACTAACATCAAACCAGAAGAGATCGAT
GGGAGAGTGGAGTTGGCTGGAACAAGGCGAGTGACAACAGCTTCAGCAGCCCAACGAA
GGGGGCGGGTAGGCAGGCACGAAGGTCGCACGGACACCTATGTGTATTCAGGACAGTG
TGACGATGATGACAGTTCACTGGTGCAGTGGAAAGAAGCGCAGATCTTGCGAGCTCGGA
GCACAAGACTGGCCTCATGGGCCTTCCGCTCACTGCCCGCTTTCCAGTCGGGAAACCTG
TCGTGCCAGCTGCATTAACATGGTCATAGCTGTTTCCTTGCGTATTGGGCGCTCTCCGCT
TCCTCGCTCACTGACTCGCTGCGCTCGGTCGTTCGGGTAAAGCCTGGGGTGCCTAATGA
GCAAAAGGCCAGCAAAAGGCCAGGAACCGTAAAAAGGCCGCGTTGCTGGCGTTTTTCCA
TAGGCTCCGCCCCCCTGACGAGCATCACAAAAATCGACGCTCAAGTCAGAGGTGGCGAA
ACCCGACAGGACTATAAAGATACCAGGCGTTTCCCCCTGGAAGCTCCCTCGTGCGCTCT
CCTGTTCCGACCCTGCCGCTTACCGGATACCTGTCCGCCTTTCTCCCTTCGGGAAGCGTG
GCGCTTTCTCATAGCTCACGCTGTAGGTATCTCAGTTCGGTGTAGGTCGTTCGCTCCAAG
CTGGGCTGTGTGCACGAACCCCCCGTTCAGCCCGACCGCTGCGCCTTATCCGGTAACTA
TCGTCTTGAGTCCAACCCGGTAAGACACGACTTATCGCCACTGGCAGCAGCCACTGGTAA
CAGGATTAGCAGAGCGAGGTATGTAGGCGGTGCTACAGAGTTCTTGAAGTGGTGGCCTA
ACTACGGCTACACTAGAAGAACAGTATTTGGTATCTGCGCTCTGCTGAAGCCAGTTACCT
TCGGAAAAAGAGTTGGTAGCTCTTGATCCGGCAAACAAACCACCGCTGGTAGCGGTGGT
TTTTTTGTTTGCAAGCAGCAGATTACGCGCAGAAAAAAAGGATCTCAAGAAGATCCTTTGA
TCTTTTCTACGGGGTCTGACGCTCAGTGGAACGAAAACTCACGTTAAGGGATTTTGGTCA
TGAGATTATCAAAAAGGATCTTCACCTAGATCCTTTTAAATTAAAAATGAAGTTTTAAATCA
ATCTAAAGTATATATGAGTAAACTTGGTCTGACAGTTACCAATGCTTAATCAGTGAGGCAC
CTATCTCAGCGATCTGTCTATTTCGTTCATCCATAGTTGCCTGACTCCCCGTCGTGTAGAT
AACTACGATACGGGAGGGCTTACCATCTGGCCCCAGTGCTGCAATGATACCGCGAGAAC
CACGCTCACCGGCTCCAGATTTATCAGCAATAAACCAGCCAGCCGGAAGGGCCGAGCGC
AGAAGTGGTCCTGCAACTTTATCCGCCTCCATCCAGTCTATTAATTGTTGCCGGGAAGCTA
GAGTAAGTAGTTCGCCAGTTAATAGTTTGCGCAACGTTGTTGCCATTGCTACAGGCATCG
TGGTGTCACGCTCGTCGTTTGGTATGGCTTCATTCAGCTCCGGTTCCCAACGATCAAGGC
GAGTTACATGATCCCCCATGTTGTGCAAAAAAGCGGTTAGCTCCTTCGGTCCTCCGATCG
TTGTCAGAAGTAAGTTGGCCGCAGTGTTATCACTCATGGTTATGGCAGCACTGCATAATTC
TCTTACTGTCATGCCATCCGTAAGATGCTTTTCTGTGACTGGTGAGTACTCAACCAAGTCA
TTCTGAGAATAGTGTATGCGGCGACCGAGTTGCTCTTGCCCGGCGTCAATACGGGATAAT
ACCGCGCCACATAGCAGAACTTTAAAAGTGCTCATCATTGGAAAACGTTCTTCGGGGCGA
AAACTCTCAAGGATCTTACCGCTGTTGAGATCCAGTTCGATGTAACCCACTCGTGCACCC
AACTGATCTTCAGCATCTTTTACTTTCACCAGCGTTTCTGGGTGAGCAAAAACAGGAAGGC
AAAATGCCGCAAAAAAGGGAATAAGGGCGACACGGAAATGTTGAATACTCATACTCTTCC
TTTTTCAATATTATTGAAGCATTTATCAGGGTTATTGTCTCATGAGCGGATACATATTTGAA
TGTATTTAGAAAAATAAACAAATAGGGGTTCCGCGCACATTTCCCCGAAAAGTGCCAC

>R1_CpG-H_M1
ATCGATAATGACGGAGTGTTGGACTCTCGTCTTTTCCTGCCGGTTACTCTCGCGGGGCCG
CGTTCAAAATACAACAGAATACCTGGATATAGCGAGCAAACGAAAGGGCCGTGGGATCAG
ACCCCTCTGAGAGTCGTTCGCGATCATTGCCCGGGAACAAGCGTGAAGATTGACAGTCA
GTGTGACAAGAGAGGAGCGTCGGTGCGAAGCACGACCGAAAGCGGGAAAATCATCCCA
GAGTGGTGCTGCCGAGCGTGTGAACTGCCACCGGTGACGTTCCGAAGCGGGACAGACT
GTTGGTATGCAATGGAGATCCGACCCGTTCACTCTCAGGGTGGCCTCGTTCGATCTATGG
TAATGGCCGACAACGGCGCGATGCTGAGCGAGGGCGGCGTCCCTGGACTCGTGGCCGT
GTTTGTTCTGATGGAGTTCTTGTTGAGAAGGCGTCCGGGTTCTGTCACATCTATTTTATGG
GGAGGTATACTCATGCTCGGTTTGCTTGTTACGGGGCTTGTAGGCGTCGAGGAAATCGTA
CGGTACTTCATCGCAGTTGGAATCACGTTCCACCTGGAGTTGGGACCAGAGATCATGGC
GCTTGTGATGCTGCAGGCCGTCTTTAGCATGCGCACATGCTATCTCACTGGGTTTCTAAC
TAAGAAGGTGATCACGACACGTGAGGTCGTGACCGTTTACTTCCTACTTTTGGTGCTTGA
GATGGGGATACCAGAAATGAACCTTGGACATTTATGGGAGTGGGCTGATTCGTTGGCGAT
GGGACTACTCATAATCAAGGCAAGCTCCATGGAAGACCGAAAAGGCTTGGGATTCCTATT
GGCTGGGCTAATGACACAGCGACACCTGACCGCCGTTCACCATGGATTGATCGTATTCCT
CACTGTTGCTTTGGCGGTGGTCGGACGAAACATCTACAACGGACAAAAAGAGAGGAAAG
GATTAAGTTTCACGATACCTTTGGCGTCTCTACTGGGCGGGCCTGGATCTGGCTTACGCA
TGTTAGCGTTGTGGGAATGCCTGAGGGGCCATGGCAGACGAAGTCTGAGTGAACCTCTA
ACTGTCGTCGGCGTCATGTTGGCAATGGCTAGTGGACTGCTGCGTCATTCTTCGCAGGA
GGCGTTACTCGTCCTCTCTGCTGGTTCATTTCTCATTCTGATGTTGATTTTGGGGACTCGC
AGACTTCAGTTGACGGCCGAATGGGCAGGATTGGCTGAGTGGAACCCTGAATTGGTGAA
CGAGGGTGGAGAGGTTAGCCTAAAAGTGCGGCAGGATGCAATGGGGAACTTGCACTTGA
CCGAAGTGGAACGCGAAGAGCGCAGGCTCGCACTGTGGCTCGTCTTTGGTTTGCTCGCT
TCCGCATTCCATTGGTCGGGAATATTGGTGACAATGGGAGCATGGACACTGTATGAGCTG
CTCTCGTCAACGCGCAGAACGGACCTTGTATTCTCGGGTCAACTGTCTGACCATGGGGA
GAGCAGGCCGTTTGACATAAAAGATGGCGTTTATCGCATATATGCGCCGGGACTCTTATG
GGGTCACC

>R1_CpG-H_M2
ATCGATAATGATGGCGTTTTGGACTCACGTCTTTTCCTGCCGGTTACGCTTGCAGGTCCG
CGTTCAAAATACAACCGAATACCCGGATATAGCGAGCAAACGAAAGGGCCTTGGGATCAG
ACGCCGCTGAGAGTCGTGCGCGATCATTGCCCGGGAACAAGCGTGAAGATTGACAGTCA
GTGTGACAAAAGAGGCGCGTCGGTTCGAAGCACGACGGAAAGCGGAAAAATAATCCCGG
AGTGGTGCTGCCGCGCGTGTGAACTGCCGCCGGTCACATTCCGCAGCGGAACAGACTGT
TGGTATGCAATGGAGATACGACCAGTTCACTCGCAGGGTGGCCTCGTTCGATCAATGGTA
ATGGCGGACAACGGCGCAATGCTGAGCGAGGGAGGCGTCCCAGGACTCGTCGCGGTTT
TTGTGCTGATGGAGTTCTTGTTGAGAAGGCGTCCTGGTTCGGTCACGTCAATTTTATGGG
GTGGTATACTCATGCTCGGTTTGCTTGTTACCGGGCTTGTCGGCGTTGAGGAGATTGTAC
GGTACTTCATCGCCGTTGGGATCACGTTCCACCTTGAGTTGGGACCTGAGATCATGGCGC
TTGTTATGCTTCAAGCAGTTTTTAGCATGCGCACATGCTATCTAACCGGGTTTCTGACCAA
GAAGGTGATCACAACGCGTGAGGTCGTGACCGTTTACTTCCTACTTTTGGTGCTTGAAAT
GGGGATACCCGAAATGAACCTTGGACATTTATGGGAGTGGGCAGATTCGTTGGCAATGG
GACTACTTATAATCAAGGCAAGTTCAATGGAAGACCGAAAAGGGTTGGGATTCCTATTGG
CCGGGCTCATGACTCAGCGACACCTGACGGCGGTTCACCATGGATTGATCGTATTCCTCA
CCGTTGCGTTGGCCGTCGTTGGACGAAACATATACAACGGACAAAAAGAGCGGAAAGGA

TTAAGTTTCACGATCCCGTTGGCGTCACTACTTGGTGGGCCGGGATCCGGCTTACGAATG
TTAGCGTTGTGGGAATGCCTTCGTGGCCATGGCAGACGAAGTCTGAGTGAACCGCTAAC
GGTCGTTGGCGTGATGTTGGCAATGGCAAGTGGACTGCTGCGTCATTCGTCTCAGGAGG
CCTTACTCGTCCTTTCCGCCGGTTCGTTTCTGATTCTGATGTTGATTTTGGGAACGCGGAG
ACTTCAGTTGACGGCAGAATGGGCGGGATTGGCAGAGTGGAACCCTGAATTGGTCAACG
AGGGTGGTGAAGTTAGCCTAAAGGTTCGGCAGGATGCAATGGGTAATTTGCACTTGACG
GAAGTCGAACGCGAAGAGCGCCGTCTCGCGCTTTGGCTCGTGTTTGGTTTGCTCGCGTC
GGCGTTCCATTGGTCAGGAATATTGGTTACAATGGGTGCGTGGACACTTTACGAGCTCCT
CTCGTCGACACGCCGAACGGACCTTGTATTCTCAGGGCAACTGTCGGACCACGGCGAGA
GCCGGCCGTTTGACATAAAAGATGGCGTCTATCGAATATACGCGCCGGGACTGTTATGG
GGTCACC

>R1_UpA-H_M1
ATCGATAATGATGGAGTCTTGGACTCTCGTCTCTTCCTGCCGGTAACTCTAGCCGGGCCC
CGTTCTAAATACAACAGAATACCTGGATACAGTGAGCAAACTAAAGGGCCGTGGGATCAG
ACCCCTCTGAGAGTGGTCCGGGACCATTGCCCGGGTACAAGCGTGAAGATAGACAGTCA
GTGTGATAAGAGAGGAGCCTCAGTCAGGAGCACCACAGAAAGCGGGAAAATTATCCCAG
AGTGGTGCTGCCGAGCTTGTGAACTGCCACCGGTGACGTTCCGTAGCGGCACTGACTGT
TGGTATGCCATGGAGATTAGGCCGGTACACTCGCAGGGTGGCCTGGTTAGATCTATGGTT
ATGGCTGACAACGGCGCTATGCTCAGCGAGGGGGGAGTCCCTGGACTAGTGGCGGTGT
TTGTCCTAATGGAGTTCTTGTTGAGAAGGCGTCCGGGTTCTGTCACCTCTATATTGTGGG
GAGGTATACTGATGCTAGGTTTGCTAGTTACAGGGCTAGTTGGAGTGGAGGAGATAGTCC
GCTACTTCATCGCAGTTGGGATAACCTTCCACCTGGAGTTGGGACCAGAGATCATGGCCC
TAGTGATGCTGCAGGCGGTCTTTAGCATGCGCACCTGCTATCTCACTGGGTTTCTCACTA
AGAAGGTAATAACCACACGTGAGGTGGTCACTGTATACTTCCTACTTTTGGTCCTGGAGA
TGGGGATACCAGAAATGAACCTAGGACATTTATGGGAGTGGGCTGATTCGTTGGCCATG
GGACTACTCATAATCAAGGCAAGCTCTATGGAAGACCGAAAAGGCTTAGGATTCCTGTTG
GCTGGGCTCATGACCCAGCGACACCTGACGGCTGTACACCATGGATTAATAGTGTTCCTC
ACTGTGGCTTTGGCCGTGGTGGGACGTAACATATATAACGGACAAAAGGAGAGGAAAGG
ATTAAGCTTCACCATCCCTTTGGCCTCGCTACTGGGGGGGCCGGGCTCTGGCTTGCGCA
TGTTAGCCTTGTGGGAGTGCCTGAGGGGCCATGGCAGACGAAGTCTAAGTGAACCCCTT
ACTGTGGTAGGGGTTATGTTAGCAATGGCTAGTGGACTGCTGCGTCATTCTTCCCAGGAG
GCTTTGCTGGTCCTCTCTGCTGGTTCCTTCCTCATACTGATGTTGATATTAGGGACCCGCA
GACTCCAGTTGACGGCGGAGTGGGCGGGATTGGCTGAGTGGAACCCTGAATTGGTAAAC
GAGGGGGGTGAGGTTAGCCTCAAGGTAAGGCAGGATGCTATGGGGAACTTGCACTTGAC
GGAAGTGGAACGGGAAGAGCGCAGGCTGGCACTCTGGCTAGTATTTGGTTTGCTGGCTT
CGGCCTTCCATTGGTCTGGAATATTGGTGACTATGGGAGCCTGGACCCTCTATGAGCTCC
TGTCGTCTACACGTAGAACTGACCTTGTATTCTCTGGGCAACTGTCCGACCACGGGGAGA
GCAGGCCTTTTGACATCAAAGATGGCGTCTATCGCATCTACGCCCCTGGACTATTGTGGG
GTCACC

>R1_UpA-H_M2
ATCGATAATGATGGGGTGTTAGACTCCCGGCTATTCCTCCCGGTTACTCTGGCCGGGCCC
CGCTCTAAATACAACAGGATCCCTGGCTATAGTGAGCAAACTAAGGGGCCGTGGGATCA
GACCCCTCTAAGAGTGGTACGGGATCATTGCCCCGGTACAAGCGTTAAGATAGACAGTCA
GTGTGACAAGAGAGGGGCCTCAGTTAGAAGCACCACTGAGAGCGGGAAAATCATCCCAG

AGTGGTGCTGCCGGGCTTGTGAACTACCACCGGTTACGTTCCGCAGCGGCACGGACTGT
TGGTATGCCATGGAGATCAGGCCGGTACACTCGCAGGGGGGCCTAGTTAGGTCTATGGT
TATGGCAGATAACGGCGCTATGCTTAGCGAGGGGGGGGTACCTGGGCTAGTAGCGGTAT
TTGTACTAATGGAGTTCTTATTGAGGAGGCGTCCGGGTTCTGTTACATCAATATTGTGGGG
AGGTATCCTTATGCTGGGGTTACTGGTTACAGGGCTGGTAGGAGTGGAGGAGATAGTAC
GCTACTTCATAGCAGTAGGCATCACCTTCCACCTAGAGTTGGGACCAGAGATTATGGCCC
TAGTTATGCTACAGGCGGTATTTAGCATGCGCACTTGCTATCTAACAGGGTTTCTAACCAA
GAAGGTAATCACCACCCGTGAGGTAGTGACTGTATACTTCCTACTGTTGGTACTGGAGAT
GGGCATACCGGAAATGAACCTAGGACATTTGTGGGAGTGGGCTGATTCGTTAGCCATGG
GACTGCTAATCATTAAGGCTAGCTCCATGGAGGACCGTAAGGGCTTGGGGTTCCTGTTGG
CAGGGCTAATGACACAGCGACACCTCACTGCAGTACACCATGGCTTAATAGTATTCCTAA
CTGTAGCTTTGGCCGTGGTAGGGCGCAACATATACAACGGGCAGAAGGAGAGGAAAGGA
TTGAGCTTCACCATACCCTTAGCCTCGCTGCTGGGGGGGCCTGGCTCAGGCTTACGCAT
GTTAGCCTTGTGGGAATGCCTTAGGGGCCATGGCAGACGTAGTCTTAGTGAACCCCTCAC
TGTGGTGGGGGTTATGTTAGCCATGGCTAGTGGTCTGCTGCGTCATTCTTCCCAGGAGG
CTTTACTGGTACTGTCTGCTGGTTCCTTTCTTATCCTTATGTTGATCTTAGGTACGCGCAG
ACTACAGTTAACGGCGGAATGGGCGGGATTAGCTGAGTGGAACCCTGAGTTGGTTAACG
AGGGGGGTGAGGTTAGCCTAAAGGTTAGGCAGGATGCCATGGGGAACTTACACTTGACG
GAGGTGGAGCGGGAGGAGCGCAGGCTAGCCCTATGGCTGGTATTTGGTTTGCTAGCCTC
GGCCTTCCATTGGTCTGGGATCTTAGTCACTATGGGAGCTTGGACACTCTATGAGCTCCT
CTCGTCCACACGCAGGACTGACCTGGTATTCTCAGGGCAACTATCCGACCACGGGGAGA
GCAGGCCTTTTGATATCAAGGATGGCGTCTATCGCATCTACGCCCCTGGCCTCTTATGGG
GTCACC

>R2_CpG-H_M1
GGTCACCGCCAGATTGGAGTCGGGTATGGAACAAAAGGAGTTCTACACACGATGTGGCA
TGTGACAAGAGGAGCCGCTCTCAGTGTGGAAGGAGCGGTTTCAGGACCATATTGGGCCG
ACGTGCGTGAGGACGTCGTTTGTTACGGAGGCGCGTGGAGCCTGGAAGGAAAATGGGG
AGGCGAAGTTGTTCAGGTCCATGCTTTTCCGCCAGACAGTGGACACAAAGTCCATCAATG
CCAACCAGGGAAACTTAATCTTGAGGGAGGGCGAGTCATGGGGGCGATCCCGATCGATC
TACCCCGTGGTACCTCAGGCAGCCCAATCATAAACGCACAAGGGATTGTGCTTGGTCTTT
ACGGTAACGGTCTGAAATCAAATGATGTTTACATCAGCAGCATCGCGCAAGGAAGCGTTG
AAAAGAGCCGTCCCGATATGCCATTGGCAGTTCAGGGGGGCAGATGGACATCGAAAGGA
AGCATCACAGTCTTGGATATGCATCCTGGATCGGGAAAGACGCATAGAGTTCTTCCCGAG
CTTATTCGTGAATGCATTGACAAAAGGTTAAGGACTGTCGTGCTTGCGCCAACGCGGGTC
GTGTTGAAAGAAATGGAAAGGGCCCTGCAAGGAAAACGTGTCAAGTTTCATTCAGCTGCC
GTCGACAACACCTCTTCGTCGTCGGGCGCGATCGTTGATGTCATGTGTCATGCCACTTAC
GTCAATCGCCGACTTTTGCCACAGGGGAGACAGAATTGGGAAGTCGCTATAATGGATGAA
GCACACTGGACGGACCCTCATAGCATAGCAGCAAGGGGTCATTTGTACTCTTTGGCGAAA
GAGAACAGATGTGCATTTGTTTTGATGACGGCCACTCCGCCGGGCAAAAGTGAAGCATTC
CCCGAATCGAAAGGAGCCATAGTTAGTGAAGAGCGACCCATTCCGGACGGGGAGTGGAG
AGATGGCTTTGATTGGGTAACGGAATTCGAAGGCCGGACCGCTTGGTTCGTGTCATCAAT
AGCCAAGGGAGGTGCGATTGCACGAACACTGAGGCAGAAAGGGAAAAGTGTGATATGTT
TGAACAGCAAGACCTTTGACAAAGATTATGGCAGAGTCCGTGAGGAAAAACCTGACTTTG
TTGTGACAACGGACATCTCAGAAATGGGGGCGAATCTCGACGTCCATAGAGTCATAGATG
GCAGGACGAACATCAAACCTGAAGAAATCGATGGACGAGTCGAGTTGGCTGGCACAAGG

CGAGTGACTACCGCATCAGCCGCGCAACGAAGGGGGCGCGTCGGAAGGCACGAAGGAC
GCACTGACACTTATGTGTATTCTGGCCAATGTGACGATGATGACAGCTCTCTCGTGCAGT
GGAAGGAAGCGCAGATCTTGC

>R2_CpG-H_M2
GGTCACCGGCAGATTGGCGTTGGTTATGGAACAAAAGGCGTTCTACACACGATGTGGCA
TGTGACGCGTGGAGCCGCGCTTAGTGTCGAAGGCGCGGTTTCGGGACCATATTGGGCC
GACGTGCGTGAGGACGTCGTTTGTTACGGAGGTGCATGGAGCCTTGAAGGAAAATGGGG
AGGTGAAGTTGTTCAGGTCCATGCGTTTCCACCAGACAGTGGACACAAAGTCCATCAATG
CCAACCGGGAAAACTTAATCTTGAAGGCGGGCGCGTTATGGGTGCGATACCAATCGACC
TACCGCGTGGTACATCGGGCAGCCCAATAATCAACGCACAAGGGATTGTTCTTGGTCTTT
ACGGTAATGGTCTGAAATCAAATGATGTTTACATAAGCAGCATTGCACAAGGAAGCGTTG
AAAAGAGCCGTCCAGATATGCCATTGGCCGTTCAGGGCGGCAGATGGACATCAAAAGGG
AGCATAACAGTTTTGGATATGCATCCCGGATCAGGAAAGACACATCGAGTCCTTCCGGAG
CTAATTCGTGAATGCATTGACAAACGGTTACGGACCGTTGTGCTCGCGCCAACACGCGTT
GTGTTGAAAGAAATGGAAAGGGCACTTCAGGGAAAACGTGTCAAGTTTCATTCAGCAGCC
GTTGACAACACGTCGTCATCATCGGGCGCGATCGTTGATGTCATGTGTCACGCGACGTAT
GTCAATCGGCGACTCTTGCCACAGGGTCGACAGAATTGGGAGGTTGCGATAATGGATGA
AGCACACTGGACCGACCCACATAGCATCGCAGCACGTGGTCATTTGTACTCATTGGCAAA
AGAGAACCGATGCGCATTTGTTTTGATGACGGCGACGCCGCCCGGAAAGAGTGAGGCAT
TCCCGGAATCAAAAGGCGCGATCGTGAGCGAAGAGCGACCGATTCCGGATGGCGAGTG
GCGCGATGGATTTGATTGGGTAACGGAATTCGAGGGGCGTACAGCATGGTTCGTTTCATC
AATCGCAAAGGGAGGTGCAATTGCACGAACACTGCGACAGAAAGGGAAAAGTGTGATAT
GTTTGAACAGCAAGACGTTTGACAAAGATTATGGACGCGTGCGTGAGGAAAAACCAGACT
TTGTTGTCACAACCGACATATCGGAAATGGGCGCGAATCTCGACGTGCATCGCGTAATAG
ATGGCAGGACGAACATCAAACCAGAAGAAATCGATGGACGAGTTGAGTTGGCAGGCACA
CGGCGTGTGACGACCGCATCGGCAGCGCAACGACGCGGACGCGTCGGACGGCATGAAG
GACGGACCGACACATATGTGTATTCAGGGCAATGTGACGATGATGACAGCTCGCTTGTTC
AGTGGAAGGAAGCGCAGATCTTGC

>R2_UpA-H_M1
GGTCACCGCCAGATAGGGGTAGGGTATGGTACAAAAGGAGTCCTACACACTATGTGGCA
TGTAACAAGAGGAGCGGCTCTCAGTGTGGAAGGAGCCGTATCAGGACCATATTGGGCCG
ACGTACGTGAGGACGTAGTCTGTTACGGGGGTGCCTGGAGCCTGGAAGGTAAATGGGGA
GGCGAAGTTGTTCAGGTCCATGCTTTTCCCCCAGACAGTGGACACAAGGTCCATCAATGC
CAACCAGGTAAACTGAATCTAGAGGGGGGGCGAGTCATGGGAGCCATCCCTATAGACCT
ACCCCGTGGTACCTCAGGCAGCCCTATCATAAACGCCCAAGGGATAGTGCTAGGTCTATA
CGGTAACGGTCTGAAATCTAATGATGTATACATCAGCAGCATTGCCCAAGGCAGCGTTGA
AAAGAGCCGTCCAGATATGCCATTAGCCGTTCAGGGGGGCAGATGGACCTCTAAAGGCA
GCATCACTGTCTTGGATATGCATCCTGGGTCGGGGAAGACACATAGAGTCCTACCCGAG
CTAATTCGGGAATGCATTGACAAGAGGTTAAGGACGGTAGTGCTGGCCCCAACGCGGGT
AGTGTTGAAAGAAATGGAGAGGGCCCTGCAGGGTAAACGTGTCAAGTTTCATTCAGCTGC
AGTAGACAACACCTCTTCATCATCAGGGGCCATAGTGGATGTCATGTGTCACGCCACTTA
TGTCAATCGCCGACTCTTACCTCAGGGGAGACAGAATTGGGAGGTAGCTATTATGGATGA
AGCCCACTGGACCGACCCTCATAGCATAGCAGCACGTGGTCATTTATACTCTTTGGCCAA
AGAGAACAGATGCGCGTTTGTGTTGATGACGGCCACTCCGCCAGGCAAGAGTGAGGCAT

TCCCTGAATCCAAAGGGGCCATAGTAAGTGAAGAGCGACCCATACCCGATGGGGAGTGG
AGAGATGGCTTTGACTGGGTGACGGAATTCGAGGGCCGGACGGCTTGGTTCGTGTCATC
TATCGCCAAGGGAGGGGCTATAGCACGTACACTAAGGCAGAAAGGTAAAAGTGTGATAT
GTTTGAACAGCAAGACCTTTGACAAAGATTATGGCAGGGTACGTGAGGAAAAACCTGACT
TTGTAGTAACTACCGATATATCTGAAATGGGGGCCAATCTGGACGTCCACAGAGTCATAG
ATGGCAGGACTAACATCAAACCTGAAGAAATCGATGGTAGAGTGGAGTTGGCTGGCACTA
GGCGAGTGACTACAGCATCAGCGGCGCAACGCAGGGGGCGGGTAGGGAGGCATGAAG
GACGCACTGACACTTATGTGTATTCTGGGCAATGTGACGATGATGACAGCTCTCTGGTAC
AGTGGAAGGAGGCGCAGATCTTGC

>R2_UpA-H_M2
GGTCACCGCCAGATAGGGGTAGGGTATGGTACAAAAGGAGTCCTACACACCATGTGGCA
TGTTACTAGAGGAGCGGCTCTTAGTGTAGAAGGAGCCGTATCAGGACCATATTGGGCCG
ACGTGCGTGAGGACGTAGTATGTTACGGGGGTGCCTGGAGCCTGGAAGGGAAATGGGG
AGGCGAAGTAGTACAGGTCCATGCTTTTCCCCCAGACAGTGGGCACAAGGTACATCAGT
GCCAACCAGGGAAGCTAAATCTGGAGGGGGGGCGAGTAATGGGGGCCATCCCTATAGA
CCTCCCCCGTGGCACCTCAGGCAGCCCTATAATCAACGCACAAGGTATAGTCCTGGGTC
TATACGGTAACGGTCTAAAATCTAATGATGTATATATAAGCAGCATAGCCCAAGGCAGCGT
AGAGAAGAGCCGTCCAGATATGCCATTAGCCGTACAGGGGGGCAGATGGACATCTAAAG
GCAGCATAACTGTATTGGATATGCATCCAGGATCGGGTAAGACCCATAGAGTCCTACCCG
AGCTCATACGTGAATGCATAGACAAGAGGTTAAGGACTGTAGTACTGGCCCCTACGCGG
GTGGTATTGAAAGAGATGGAGAGGGCCCTGCAGGGGAAACGGGTTAAGTTTCATTCAGC
TGCCGTAGACAACACCTCCTCATCCTCTGGGGCCATAGTAGATGTAATGTGTCACGCCAC
TTATGTGAATCGCCGACTCTTACCACAGGGGAGACAGAATTGGGAGGTAGCTATAATGGA
TGAAGCCCACTGGACCGACCCTCATAGCATAGCCGCACGTGGTCATTTGTACTCTTTGGC
CAAGGAGAACAGATGCGCGTTTGTCTTGATGACGGCCACGCCGCCAGGCAAGAGTGAGG
CATTCCCTGAGTCCAAAGGAGCCATAGTTAGTGAGGAGCGACCCATACCCGATGGGGAG
TGGAGAGATGGCTTTGATTGGGTAACGGAGTTCGAGGGCCGTACGGCTTGGTTCGTCTC
CTCTATAGCCAAGGGAGGTGCCATAGCACGTACCCTTAGGCAGAAAGGTAAAAGTGTTAT
ATGTTTAAACAGCAAGACCTTTGACAAAGATTATGGCAGAGTACGTGAGGAGAAACCTGA
CTTTGTGGTTACTACCGACATCTCAGAAATGGGGGCCAATCTAGACGTCCATAGGGTAAT
AGATGGCAGGACTAACATCAAACCTGAAGAGATAGATGGGAGGGTAGAGTTGGCTGGCA
CTAGGCGAGTTACTACTGCCTCAGCGGCGCAACGCAGGGGGCGGGTAGGTAGGCATGA
AGGACGCACTGACACTTATGTATATTCTGGGCAATGTGACGATGATGACAGCTCTCTAGT
GCAGTGGAAGGAAGCGCAGATCTTGC
